# Flooding Causes Dramatic Compositional Shifts and Depletion of Putative Beneficial Bacteria on the Spring Wheat Microbiota

**DOI:** 10.3389/fmicb.2021.773116

**Published:** 2021-11-05

**Authors:** Davide Francioli, Geeisy Cid, Saranya Kanukollu, Andreas Ulrich, Mohammad-Reza Hajirezaei, Steffen Kolb

**Affiliations:** ^1^Microbial Biogeochemistry, Research Area Landscape Functioning, Leibniz Center for Agricultural Landscape Research e.V. (ZALF), Müncheberg, Germany; ^2^Department of Physiology and Cell Biology, Leibniz Institute of Plant Genetics and Crop Plant Research, Gatersleben, Germany; ^3^Faculty of Life Sciences, Thaer Institute, Humboldt University of Berlin, Berlin, Germany

**Keywords:** plant-microbe interactions, flooding, metabarcoding, bacteria, spring wheat, plant traits

## Abstract

Flooding affects both above- and below-ground ecosystem processes, and it represents a substantial threat for crop and cereal productivity under climate change. Plant-associated microbiota play a crucial role in plant growth and fitness, but we still have a limited understanding of the response of the crop-microbiota complex under extreme weather events, such as flooding. Soil microbes are highly sensitive to abiotic disturbance, and shifts in microbial community composition, structure and functions are expected when soil conditions are altered due to flooding events (e.g., anoxia, pH alteration, changes in nutrient concentration). Here, we established a pot experiment to determine the effects of flooding stress on the spring wheat-microbiota complex. Since plant phenology could be an important factor in the response to hydrological stress, flooding was induced only once and at different plant growth stages (PGSs), such as tillering, booting and flowering. After each flooding event, we measured in the control and flooded pots several edaphic and plant properties and characterized the bacterial community associated to the rhizosphere and roots of wheat plant using a metabarcoding approach. In our study, flooding caused a significant reduction in plant development and we observed dramatic shifts in bacterial community composition at each PGS in which the hydrological stress was induced. However, a more pronounced disruption in community assembly was always shown in younger plants. Generally, flooding caused a (i) significant increase of bacterial taxa with anaerobic respiratory capabilities, such as members of Firmicutes and Desulfobacterota, (ii) a significant reduction in Actinobacteria and Proteobacteria, (iii) depletion of several putative plant-beneficial taxa, and (iv) increases of the abundance of potential detrimental bacteria. These significant differences in community composition between flooded and control samples were correlated with changes in soil conditions and plant properties caused by the hydrological stress, with pH and total N as the soil, and S, Na, Mn, and Ca concentrations as the root properties most influencing microbial assemblage in the wheat mircobiota under flooding stress. Collectively, our findings demonstrated the role of flooding on restructuring the spring wheat microbiota, and highlighted the detrimental effect of this hydrological stress on plant fitness and performance.

## Introduction

Climate change has increased the frequency and magnitude of extreme weather events such as drought, floods, heat waves and wildfires (Cox et al., 2002; Cook et al., 2018; Di Virgilio et al., 2019). These extreme weather events significantly impact on plants and soil biota and, in turn, they affect global biogeochemical cycling and ecosystem services, such as crop plant productivity in farming systems (Schröter et al., 2005; Vanbergen, 2013). It has been predicted that an increased number of incidences of extreme precipitation events will be a result of global warming, which will lead to an increased flooding frequency (Bevacqua et al., 2019; Konapala et al., 2020). Crop fitness and productivity are negatively impacted by water inundation (Bailey-Serres et al., 2012). Under flooding stress, crop yield can be highly reduced (Rhine et al., 2010; Morton et al., 2015; Ding et al., 2020) and even short-term events of few days can significantly affect wheat growth (Malik et al., 2002). Wheat yield losses due to flooding might range from 10 to over 50% (Jincai et al., 2001; Kaur et al., 2020; Tian et al., 2021), which however, depends on waterlogging duration, wheat genotype, growth stage, soil type and agricultural management.

Changes in belowground environments following flooding events are no less important than those that occur aboveground. Plant-associated microbiota plays a key role in fostering the host plant fitness (Turner et al., 2013; Compant et al., 2019), and it is well established that its composition is influenced by many host-associated and environmental factors (Francioli et al., 2016; Eisenhauer and Powell, 2017; Leff et al., 2018; Lewin et al., 2021). Recent research has demonstrated that alteration in soil moisture have significant effects on soil and root-associated microorganisms (Naylor and Coleman-Derr, 2018; Xu et al., 2018; Francioli et al., 2020). Many features of bacterial community, such as biomass, composition, structure and community assembly processes are sensitive to the hydrological regime in soil (Argiroff et al., 2017; Gschwend et al., 2020; Shen et al., 2021). Flooding results in changes of the osmotic activity and promotes oxygen depletion, fostering anoxia and anaerobes able to thrive under such conditions (Schimel et al., 2007). Microbial traits associated with resistance and resilience to hydrological stress may include endospore formation, production of osmoprotectants and specific cell wall structures, as well as different energy metabolisms (e.g., anaerobic and facultative respiration, fermenation, microaerophily; Unger et al., 2009; Bardgett and Caruso, 2020). Such physiological traits might be associated with specific taxa or bacterial clades such as the Gram-positive phylum Firmicutes and its endospore-forming members, e.g., *Bacillus* and *Clostridium*. These traits tend to be conserved at different phylogenetic levels (Martiny et al., 2015). Nonetheless, the response of the soil microbiota on flooding and waterlogging associated with typical crop plant hosts are largely unexplored.

Moreover, indirect effects of hydrological regimes on the plant-associated microbiota can be mediated through plant and soil physicochemical factors. Anoxia resulting from flooding can profoundly influence plant growth and, thus, indirectly altering soil bacterial microbiota through changes in the quality and quantity of rhizodeposits including exudates, competition for nutrients, or other mechanisms (Henry et al., 2007; Hartman and Tringe, 2019). In addition, soil undergoes many physiochemical changes in response to oversaturation by flooding. Several soil physicochemical variables, such as pH, nutrient concentrations, and redox status, are tightly linked to water availability, providing certain possible mechanisms for the association among water regime, edaphic properties and soil bacterial community compositions (Pett-Ridge and Firestone, 2005; Wilson et al., 2011; Moche et al., 2015).

Considering that the frequencies and intensity of extreme precipitation events are predicted to increase over the upcoming years, it is necessary to understand how these environmental changes will affect the composition and biodiversity of the microbiota associated with crop plants in agroecosystems. Thus, we setup a pot experiment to investigate the effect of flooding on the wheat plant-microbiota. Using a metabarcoding approach, we monitored responses of the bacterial community associated with different wheat compartments (rhizosphere and root) to flooding stress. Since plant phenology is an important driver in plant microbiota assembly (Donn et al., 2015; Francioli et al., 2018), and abiotic stress might affect differentially the plant-microbiota complex depending on the specific plant growth stage (PGS) in which it occur (Na et al., 2019; Breitkreuz et al., 2020), we imposed flooding stress only once, either at tillering, booting or flowering. Moreover, several soil and plant traits were measured through the whole experiment to correlate edaphic and physiological plant changes with shifts in bacterial community assemblage. We hypothesized that (1) soil physio-chemistry and plant traits will be strongly influenced by flooding, thus correlated indirectly with shifts in bacterial microbiota structure. (2) The bacterial microbiota will be differentially affected by the timing of flooding events, with early microbiota development being more susceptible to disruption. (3) The general response of the bacterial microbiota to flooding will show a plant phylogenetic signals together with shifts in abundance of important ecologically taxa.

## Materials and Methods

### Experimental Setup

The response of the wheat microbiota complex to flooding stress was investigated in a pot experiment that was conducted from September to December 2019. The experiment was carried out in a greenhouse at the Leibniz Institute of Plant Genetics and Crop Plant Research IPK-Gatersleben, Germany. Seeds of Chinese spring wheat (*Triticum aestivum* L.) were germinated in sieved soil (2 mm), which was obtained from the experimental station in Dedelow (Germany). The soil was a loamy sandy/medium silty sandy soil (S3/Su3 according to the German texture classification; Ad-hoc-AG-Boden, 2005) and its physiochemical properties are reported in Supplementary Table 1.

Seeds were germinated under controlled conditions and optimal watering. After the third leaf had appeared, i.e., 3 weeks after sowing, seedlings were individually transferred to 10L-pots containing 5 kg of the same soil used for germination (one seedling per pot). Tillering stage was initiated under controlled conditions of day/night temperature, i.e., 18/16°C, air humidity 70%, light intensity 250–300 μmol/(m^2^⋅s) and photoperiod of 16 h light/8 h darkness. Pots were placed on tables in the greenhouse in a complete randomize design. Zadoks scale (Zadoks et al., 1974) was considered to monitor the developmental stage of the plants and the application of flooding. Flooding stress was induced only once and for a period of 12 day, at either tillering, booting or flowering and replicates were destructively sampled (Supplementary Figure 1). Previous studies on different soil types have reported that complete oxygen depletion in the top soil occurs within 2–8 days of flooding (Cannell et al., 1980; Meyer et al., 1985; Drew, 1992). Since the aim of the experiment was to investigate the response of the soil-wheat-microbe complex to a severe hydrological stress, we applied flooding for 12 days in order to be sure that oxygen was depleted in the flooded treatment. We established six replicates for each combination of PGS and water treatment, for a total of 36 pots. Control and flooding treatments were arranged in parallel considering the water holding capacity (WHC) of the soil used for the experiment. The WHC of the soil used was determined by a pre-experiment prior the set-up of the main experiment. Five individual pots containing the same amount of soil (5 kg) were weighed, over-watered and left draining overnight. The weight of the pots was registered next day and considered as WHC. Control plants were monitored at 50% WHC, which was in correspondence with the field capacity of the soil. Flooding was established by keeping water approximately 5 cm above the soil surface during the 12 days period.

### Plant and Soil Sampling

After 12 days under flooding, plants of the control and flooding treatments were harvested, and tillers and spikes number documented. The material of interest (soil rhizosphere and plant tissues) used for further analysis was harvested from the same plants using the following procedure. Shoots and roots were separated, and their fresh weight was immediately measured. Rhizosphere soil, defined as soil which remained attached to the roots after the plant had been uprooted and shaken (Katznelson et al., 1948) was collected in sterile zipbags. Afterward, roots were carefully washed with tap water removing as much as possible of the remaining soil particles. Soil and root samples for molecular analysis were immediately frozen and stored at −80°C. Several macro and micronutrients concentrations in the roots were measured. Nitrogen (N) and carbon (C) concentration were analyzed in 1.5 mg ground powder by a EuroEA3000 (EuroVector SpA, Redavalle, Italy) using software version Callidus 5.1 (Muñoz-Huerta et al., 2013). For calibration the standard 2,5-Bis(5-tert-butyl-benzoxazol-2-yl) thiophene with 72.52% carbon and 6.51% nitrogen from HEKAtech GmbH (Wegberg, Germany) were used. A sector field high-resolution mass spectrometer (HR)-ICP-MS (Element 2, Thermo Fisher Scientific, Germany) was employed to measure P, Mg, S, K Ca, Mn, Zn, and Na concentrations in the root. The following edaphic properties were measured from the soil samples. Total organic carbon (TOC) and total nitrogen (TN) contents were determined in triplicate by dry combustion using a Vario EL III C/H/N analyzer (Elementar, Hanau, Germany). Since the carbonate concentration of the soils was negligible (<2%), the total C concentration measured was considered to represent TOC. Plant available P was extracted from fresh soil with double lactate (1:50 w/v, pH 3.6, 1.5 h; Riehm, 1943). After filtration of the suspension (Whatman Schleicher and Schuell 595 1/5 Ø 270 mm), the extracted P was quantified colorimetrically using the molybdenum blue method (Murphy and Riley, 1962). Mn, Ca, Na, K, Mg, concentration in soil were measured using an inductively coupled plasma-optical emission spectrometry-ICP-OES (ICP-iCAP 6300 DUO, Thermo Fisher Scientific, Germany).

### DNA Extraction, Amplicon Library Preparation, and Sequencing

DNA was extracted from the soil and root samples collected using the DNeasy PowerLyzer PowerSoil Kit (Qiagen). Bacterial DNA amplification was performed using the primers 799f (Chelius and Triplett, 2001) and 1193r (Bodenhausen et al., 2013) following the PCR protocol described previously (Francioli et al., 2021a). The amplicons were sequenced on an Illumina MiSeq instrument with 2 × 300 base pair kits by LGC genomics GmbH, Berlin, Germany. Demultiplexing was performed with Illumina bcl2fastq 2.17.1.14 software following clipping of barcode and sequencing adapters. Primer were removed using Cutadapt v3.0 (Martin, 2011) following sequence processing using QIIME 2 v2020. Amplicon sequence variants (ASV; also known as zero-radius operational taxonomic units; Callahan et al., 2017) were determined from raw sequence data using the DADA2 pipeline (Callahan et al., 2016). Only ASVs that were detected in more than two samples were included in the data analyses. Alpha diversity metrics were calculated from the normalized sequence library, which was rarefied to 20,000 reads per sample. For taxonomic assignment of the ASVs, the representative sequences were classified using the naïve Bayesian classifier for Silva 138. Non-bacterial ASVs were discarded. All sequences have been submitted to the European Nucleotide Archive (study accession number PRJEB47399).

### Statistical Analyses

Univariate Analysis of Variance (ANOVA) followed by Tukey’s honestly significant difference (HSD) *post hoc* test was used to test for differences in soil and plant properties, among the treatments and PGS. All the variables were tested for normality using Shapiro-Wilk and Jarque-Bera tests and the equality of group variances was examined using Levene’s test. A log10 transformation was applied to all variables that did not meet the parametric assumptions. Correlation among the soil and plant traits were determined using Spearman’s rank correlation. Environmental variables with a Spearman rank correlation coefficient *p* > 0.8 were removed and excluded from further analysis. Effects of soil-plant compartment, PGS and watering treatment on the bacterial richness were tested by univariate PERMANOVA models (Anderson, 2017). Pairwise differences in bacterial ASV richness between water treatment at the same PGS and compartment were estimated using ANOVA followed by a Tukey’s HSD *post hoc* test. All the phylogenetic analyses were performed using the package “picante” (Kembel et al., 2010). First, a phylogenetic distance matrix based on a maximum-likelihood 16S rRNA tree was generated in QIIME2 with FastTree2 (Price et al., 2010). The phylogenetic distance matrix was used to calculated (i) standardized effect size of phylogenetic diversity (ses.PD), (ii) the net relatedness index (NRI), and (iii) the nearest taxa index (NTI). ses.PD was calculated by comparing the observed phylogenetic diversity (PD) value from the mean of the null distribution (999 null iterations) based on random shuffling of ASV labels across the phylogenetic tips. Negative ses.PD values and low quantiles (*p* < 0.05) indicated that co-occurring species are more closely related than expected by chance (clustering), whereas positive values and high quantiles (*p* > 0.05) indicate that the co-occurring species are less closely related than expected by chance (overdispersion) (Webb et al., 2002). NRI was calculated using the “standardized effect size of pairwise distances in communities” function (*ses.mpd*) and the nearest taxa index (NTI) using the “standardized effect size of mean nearest taxon distances” function (*ses.mntd*). NRI is a measure of the mean relatedness between members of microbial communities, and the NTI is a measure of the smallest mean phylogenetic distance between all pairs of n taxa in a community sample. For both NRI and NTI analyses, the null model was randomized 999 times and set to “phylogeny.pool,” which randomly draws species from the phylogeny for its null distribution. NRI and NTI were calculated for bacterial microbiota from each soil-plant compartment, PGS and watering treatment. Positive NRI/NTI values indicate a microbiota of which taxa are on average more closely related to one another than they are to members of the randomized (null model) microbial species pool. To statistically compare ses.PD, NRI and NTI values for each type of sample (compartment, PGS, watering treatment), we used one-way ANOVA followed by a Tukey’s HSD *post hoc* test. Differences in the bacterial microbiota structure across plant-soil compartment, PGS and flooding treatment, we first calculated Bray-Curtis dissimilarities using square-root transformed relative abundances (Hellinger transformation; Legendre and Gallagher, 2001). Permutational multivariate analysis of variances (PERMANOVA) based on the Bray-Curtis dissimilarity index was performed to analyze the effect of the above mentioned experimental factors on the bacterial community structure, using 999 permutations for each test. Similarities in the bacterial community structure among the control and flooded treatments at each PGS were investigated using Analysis of Similarity (ANOSIM) algorithm. Furthermore, we performed variance partitioning based on redundancy analysis (RDA) to quantify the contribution of soil and root properties, PGS and water treatment to the structure of the bacterial community in each compartment. Following Blanchet et al. (2008) we first tested the significance of the global model using all predictors. Variable selection was then performed using forward selection implemented with function *forward.sel* in the R package “packfor” (Dray et al., 2011). Variance partitioning was conducted using the *varpart* function in the “vegan” R package (Oksanen et al., 2018). Then a model of multivariate analysis of variance was constructed using distance-based redundancy analysis (db-RDA) based on the Bray-Curtis distance to determine the environmental variables that were most influential on the bacterial community structure within each compartment. Ternary plots were performed using the package “microbiomeutilities.” Linear discriminant analysis effect size (LEfSe) (Segata et al., 2011) was applied to identify biomarker taxa explaining differences between the bacterial microbiota across PGS in the control treatment, and also between flooded and control samples at each PGS in both plant-soil compartments. All the data were analyzed with R version 3.6 (R Core Team, 2014).

## Results

We conducted a pot experiment with the model crop plant spring wheat *Triticum aestivum* cv. *Chinese Spring* to assess the impact of flooding stress on the structure of the bacterial microbiota associated to the rhizo- and endosphere. As plant phenology has been implicated in shaping the plant microbiota and abiotic stress might have a differential impact on the crop microbiota complex depending on the specific PGS, wheat plants were subjected to a period of 12 days of flooding either at tillering, booting and flowering. After such a period, stressed plants were harvested and removed from the experiment. A variety of soil, plant and microbiota related traits were measured to reveal the response of the wheat microbiota complex (Supplementary Figure 1).

### Plant Performance and Soil-Plant Properties in the Different Watering Treatments and Across Plant Growth Stages

Flooding had a significant effect on plant growth, as well as on soil and plant properties. At tillering, we observed a significant reduction (*p* < 0.05) only in root biomass (55%), but flooding at booting caused shoot and root dry biomass decreases by 25 and 70%, respectively, compared to control plants (Supplementary Figure 2). No change in above- and belowground biomass was observed when the flooding stress occurred at flowering. Wheat plants exposed to flooding stress developed 29% less tillers and a showed a decrease of 18% in the spike to tiller ratio (Supplementary Figure 2).

Flooding had a strong influence on edaphic parameters. In general, a significant increase (*p* < 0.05) in soil moisture, pH, Zn, and available P was observed in flooded soil at all developmental stages (Supplementary Figure 3). Furthermore, significant increases (*p* < 0.05) of total soil C and S occurred at tillering, whereas a significant increase (*p* < 0.05) of the total N content occurred at flowering in the flooding treatments. At root level, flooding caused a significant decrease (*p* < 0.05) of the concentration of Mg, S, and Ca during tillering (Supplementary Figure 4) and booting. We also found a significant increase (*p* < 0.05) of the Mn content under flooding, which was two times higher compared to the control (Supplementary Figure 4). In the control plants, we observed shifts in soil and root nutrient concentrations across the different PGS. For example, a significant increase (*p* < 0.05) of soil total C, total S and root S and Na was observed at flowering, while the concentration of soil total N and root P was significantly higher (*p* < 0.05) at tillering and booting.

### Bacterial Richness and Biodiversity

A total of 5,318,073 bacterial 16S rRNA gene high quality reads were recovered from all the samples, which clustered in 6241 bacterial ASVs. Overall, bacterial sequences were affiliated to 31 phyla, 89 classes, 194 orders, 325 families, and 630 genera. Proteobacteria was the most abundant phylum, comprising approximately 41% of the reads across all samples (1661 ASVs), followed by Actinobacteriota (30.5% of reads; 1444 ASVs) and Firmicutes (10% of reads; 636 ASVs). A small proportion of members of the Bacteroidota (6.5%, of reads; 496 ASVs), Chloroflexi (2.3% of reads; 425 ASVs), and Myxococcota (2.1% of reads; 315 ASVs) was also detected.

**FIGURE 1:**
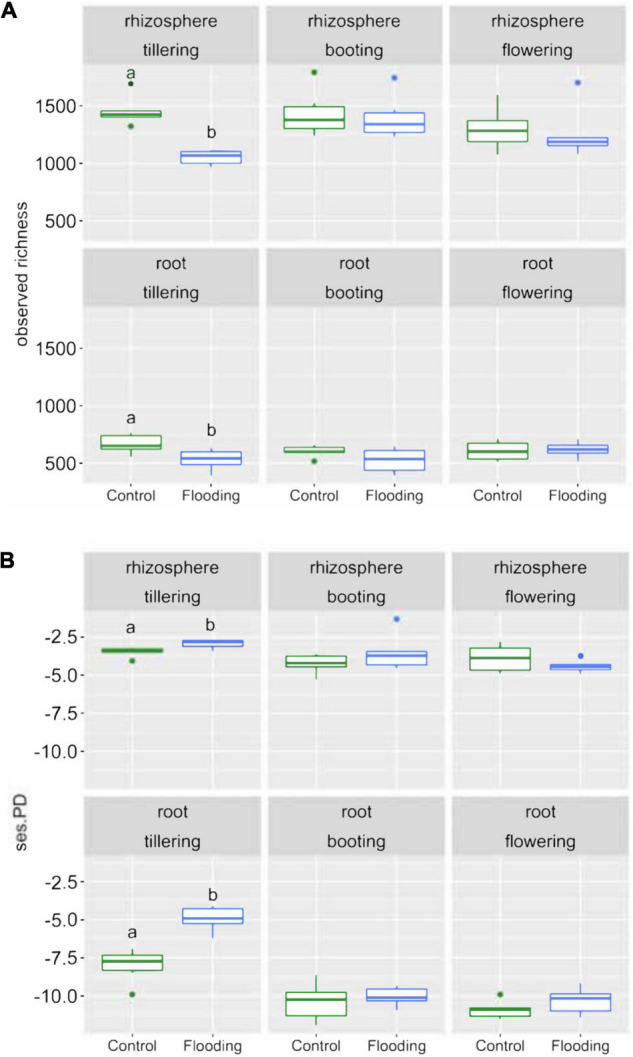
Box plots of the observed richness **(A)** and standardize effect size of phylogenetic diversity (ses.PD) **(B)** of the bacterial communities in the samples studied. Different letters indicate significant differences between water treatments within the same PGS based on Tukey’s HSD test *p* < 0.05.

We calculated bacterial richness and several phylogenetic metrics to assess how bacterial alpha-diversity was influenced by soil-plant compartment, PGS and watering treatment. Analysis of variance revealed that the soil-plant compartment had the greatest effect on bacterial richness that significantly (*p* < 0.05) decreased from rhizosphere to the root (Supplementary Table 2 and Figure 1A). Watering treatment had a marginal but significant effect (*p* < 0.05) on bacterial richness, while PGS did not. A significant interaction (*p* < 0.05) between PGS and watering treatment was detected. This latter result was evident when looking at each combination of plant-soil compartment and PGS, since most of the differences in alpha diversity were found at tillering, with significant lower values in the flooded than in the control samples, irrespective of compartment (Figure 1A).

Phylogenetic relatedness among the bacterial communities within our samples was evaluated based on the standardized effect size of phylogenetic diversity (ses.PD). Overall, the bacterial communities were relatively phylogenetic clustered, but significantly lower (*p* < 0.05) values in phylogenetic similarity were always found at tillering compared to later PGS (Figure 1B). Remarkably, the bacterial communities associated with flooded samples were characterized at tillering by the lowest effect size of phylogenetic diversity. On the contrary, at booting and flowering we observed no significant differences in phylogenetic diversity regardless of watering treatments and PGS. We also calculated NRI and NTI parameters, which measure both alternative aspects of community phylogenetic clustering vs. overdispersion. These results confirmed the findings obtained by the ses.PD analysis (Supplementary Figure 5).

### Effect of Compartment, Flooding, and PGS on Bacterial β-Diversity

**FIGURE 2:**
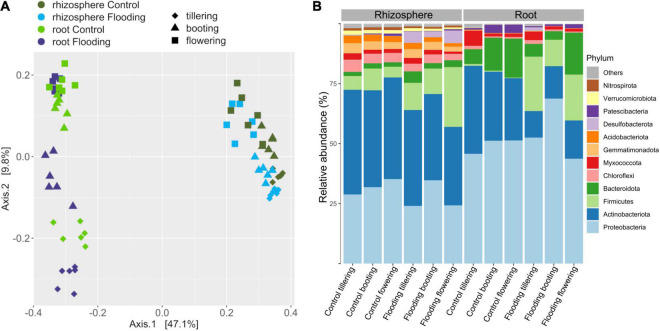
Principal coordinates analysis (PCoA) Bray-Curtis dissimilarity metrics **(A)** and mean relative abundance of the main phyla **(B)** of the bacterial communities detected in this study.

Factors that were responsible in shaping the microbiota structure between samples were firstly explored by beta-diversity employing a principal coordinate analysis with Bray-Curtis dissimilarities (Figure 2). Altogether, the two axes accounted for 55.4% of the variance, with the first coordinate (47.1%) primarily discriminating the samples by compartments, while along the second coordinate (9.8%) a separation by PGS, which was more pronounced in the root samples, was observed. Principal coordinate analysis based on weighted UniFrac distance, which accounts for phylogenetic relationships among microbial taxa, confirmed the discrimination between compartments along the first coordinate, but it also displayed along the second axis a clear separation between the bacterial communities of the flooded and control samples (Supplementary Figure 6). This latter result suggested that flooding stress induced a phylogenetic compositional response in the bacterial assemblage dynamics at each PGS it was induced. PERMANOVA analysis on the full bacterial ASV dataset confirmed that rhizosphere and roots were characterized by distinct bacterial microbiota (Table 1). The large differences in community structure between the two soil-plant compartments were primarily due to the different composition of their specific microbiota, as more than half of the ASVs that were solely found in the rhizosphere, whereas most of the root-associated bacterial ASVs were also detected in the rhizosphere (Supplementary Figure 7). The root microbiota was dominated by Proteobacteria, Patescibacteria, Myxococcota, and Bacteroidota, while the rhizosphere was significantly enriched (*p* < 0.05) in Actinobacteria, Chloroflexi, Desulfobacteriota, Acidobacteria, and Verrucomicrobiota (Figure 2B and Supplementary Figure 7). Interestingly, bacterial members of the latter two phyla were completely depleted in root samples, further indicating a strong niche compartmentalization effect promoted by the plant host (Figure 2B and Supplementary Figure 7).

**TABLE 1:** The relative importance of soil-plant compartment, plant growth stage (PGS) and watering treatment (WT) for the total bacterial community structure associated to the samples investigated in this study as revealed by PERMANOVA.

Parameter	df	Pseudo-F	*R* ^2^	*P*−value
Compartment	1	105.279	0.4638	0.001
PGS	2	11.534	0.1016	0.001
WT	1	12.611	0.0690	0.001
Compartment * PGS	2	4.263	0.0375	0.002
Compartment * WT	1	4.365	0.0192	0.007
PGS * WT	2	4.283	0.0377	0.003
Compartment * PGS * WT	2	105.279	0.4638	0.001

**FIGURE 3:**
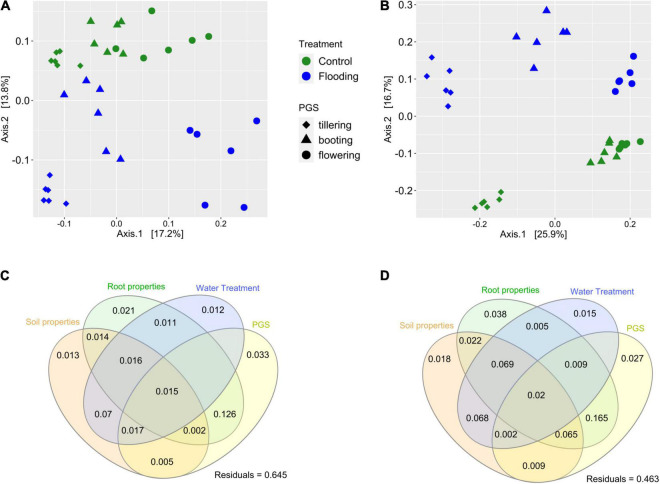
Principal coordinates analysis (PCoA) of the bacterial communities detected in the rhizosphere **(A)** and in the root **(B)** compartment. Variation partitioning analysis, illustrating the effects soil parameters, root traits, watering treatment and plant growth stage (PGS) in the community for the rhizosphere **(C)** and root **(D)** compartment. Each ellipse represents the portion of variation accounted by each factor. Shared variance is represented by the intersecting portions of the ellipses. Values < 0 are not shown. Unexplained variation (residuals) is reported in the plot.

Within each compartment, substantial and significant effects of flooding and plant phenology on the bacterial microbiota structure were found. In the rhizosphere, PERMANOVA analysis revealed that flooding explained 11.4% of variance, PGS 20.5% and the interaction of these two terms accounted for an additional 9.8% of variation (Table 2). However, a higher influence of the experimental treatments on the bacterial microbiota was found in the root compartment, as flooding explained 16.8% of variance, PGS 30% and the interactions of these two factors captured a further 14.5%, for a total of 61% of variation (Table 2). Principal coordinates analysis on the bacterial microbiota of the rhizosphere and roots confirmed these results, distinguishing the samples associated with a particular PGS along the first axis, while the flooded samples where clearly separated from the control by the second axis (Figures 3A,B). ANOSIM provided further evidence that the bacterial microbiota of the flooded wheat plants were significantly different (*p* < 0.05) compared to the control at each PGS (Supplementary Tables 3, 4).

**TABLE 2:** The relative importance of plant growth stage (PGS) and watering treatment (WT) for the total bacterial community structure associated to rhizosphere and root compartments in the samples investigated in this study as revealed by PERMANOVA.

	Rhizosphere				Root			
Parameter	df	Pseudo-F	*R* ^2^	*P*−value	df	Pseudo-F	*R* ^2^	*P*−value
PGS	2	5.2378	0.2041	0.001	2	11.7182	0.3010	0.001
WT	1	5.8123	0.1132	0.001	1	13.1147	0.1684	0.001
PGS * WT	2	2.5079	0.0977	0.001	2	5.6546	0.1452	0.001

Variance partitioning was conducted to quantify the contribution of edaphic and root properties, and their interactions with watering treatment and PGS on the structure of the bacterial microbiota. In both compartments, these four experimental factors captured together a large proportion of the variance, accounting for 35 and 54%, in the rhizosphere and root, respectively (Figures 3C,D). In general, the pure effect of these variables on the bacterial community structure was marginal, since most of the variance explained by them was shared, indicating an interactive effect of PGS and watering treatment on the root and soil properties, which in turn significantly affected the bacterial microbiota assemblage. A significant effect (*p* < 0.05) of various soil and root properties on the microbiota was further revealed by partial db-RDA. Soil pH and total N, together with root S content were significant factors affecting the bacterial microbiota structures in both compartments (Supplementary Table 5). In addition, the root microbiota was also significantly correlated with root K, Na, Mn, and Ca concentrations (Supplementary Table 5).

**FIGURE 4:**
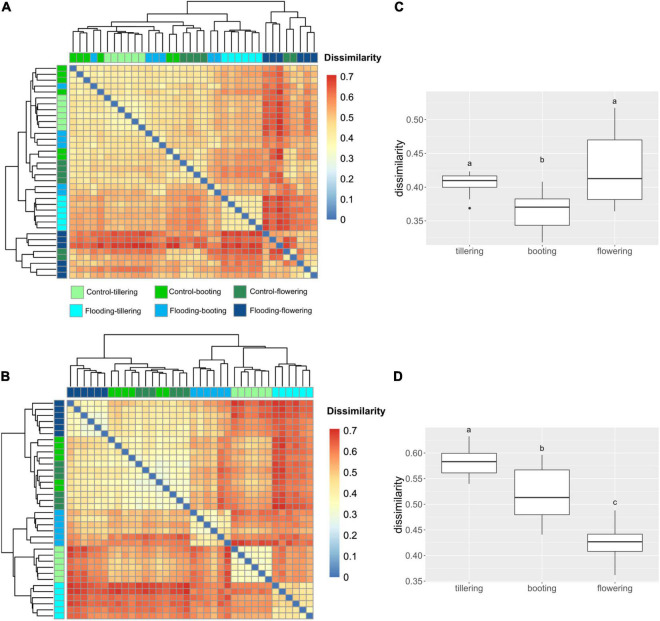
Heatmap representing the Bray-Curtis dissimilarity in bacterial community structure between watering treatment and plant growth stages in the rhizosphere **(A)** and root **(B)**. Box plots of Bray-Curtis dissimilarity index between watering treatment and plant growth stages in the rhizosphere **(C)** and root **(D)**. The different letters indicate significant differences among plant growth stages (Tukey’s HSD test *p* < 0.05.).

We compared structural dissimilarities of the bacterial microbiota between flooding and control treatments at each PGS to resolve at which PGS the application of flooding had the largest effect. In the rhizosphere, the largest impact of flooding stress on the bacterial microbiota structure was observed in the earliest (tillering) and latest stage (flowering) of the plant growth. While in the roots, the greatest impact of flooding was found at tillering, followed by booting and flowering (Figure 4). It is noteworthy to mention, that dissimilarities between flooding and control samples were always lower in the soil than in the root. This observation confirms that the influence of flooding was more pronounced on the root-associated bacterial microbiota.

### Dynamics of the Bacterial Microbiota Over Plant Growth Stages

**FIGURE 5:**
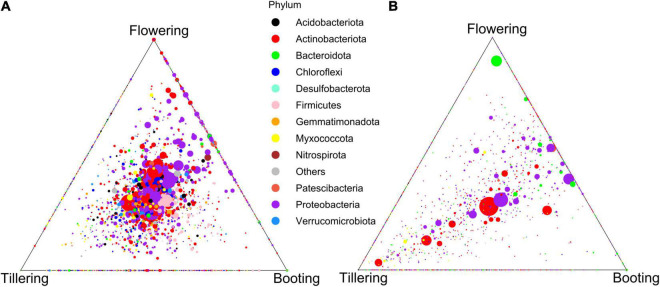
Ternary plot of the ASVs distribution across plant growth stages in the rhizosphere **(A)** and in the roots **(B)** of the control plants.

To better understand the effect of watering treatment in the rhizosphere and root microbiota, we sought to explore the temporal dynamics of the bacterial communities under control condition. β-diversity changes in response to PGS were revealed in both compartments, with the bacterial communities at booting and flowering stages always being more similar among each other than with the one at tillering (Figures 4, 5 and Supplementary Figure 8). However, higher microbiota dissimilarities between early (tillering) and late PGS (booting, flowering) were found in the root compartment than in the rhizosphere (Figures 4, 5 and Supplementary Tables 3, 4). This indicated a stronger plant phenological effect on the endospheric and rhizoplan bacteria. This result was also reflected by variance partitioning analysis, that revealed that the pure and interactive effects captured by PGS and root traits were relatively higher in roots than in the rhizosphere (Supplementary Figure 9), suggesting that changes of root traits over time were highly determinant for the root microbiota assembly.

We found that the observed differences in bacterial microbiota over PGS were primarily due to shifts in the abundance of taxa shared across all three PGS. Indeed, PGS-exclusive ASVs accounted for a marginal proportion of sequences (ranging from 1.2 to 5.7%), while more than half of the ASVs detected were found in all PGS and accounted for more than three quarters of the total sequences detected in the control treatment (Supplementary Figure 10). Many shared ASVs were affiliated with several representative genera (>1% abundance), most of which showed abundance shifts across PGS (Supplementary Figure 10).

Dominant bacterial phylotypes as indicative biomarkers at each PGS were identified by linear discriminant analysis (LDA) effect size (LEfSe) (Supplementary Figure 11). In the rhizosphere, members affiliated with the phyla Chloroflexi, Acidobacteria, Gemmatimonadota, and Myxococcota were significant more abundant (*p* < 0.05) at tillering than in other PGS. On the other hand, bacterial taxa within phyla Proteobacteria, Actinobacteria, and Patescibacteria were found significantly more abundant (*p* < 0.05) at flowering, while booting stage showed a significant higher proportion (*p* < 0.05) of Firmicutes. LEfSe analysis identified *Gaiella*, *Streptomycetes*, *Mycobacterium*, MB-A2_108 at tillering, *Bacillus* and *Penibacillus* (both belonging to the class Bacilli) and *Nitrospira* at booting, and *Sphingomonas*, *Massilia*, *Mesorhizobium*, and *Arthrobacter* at flowering as biomarker genera. In the roots, contrarily to the rhizosphere, Actinobacteria, Firmicutes, Fibrobacterota, and Myxococcota phyla were found as biomarker at tillering stage, Proteobacteria at booting, while Bacteroidota and Patescibacteria were enriched at flowering (Supplementary Figure 10). Indeed, the roots of young plants were mainly enriched in several genera affiliated with Actinobacteriota, i.e., *Streptomyces*, *Kribbella* and *Lechevalieria*. The most discriminant biomarker taxa at booting were the Proteobacteria families *Devosiaceae*, *Rhizobiaceae*, *Pseudomonadaceae*, and *Caulobacteraceae*, whereas at flowering they were represented by species of the genus *Flavobacterium* (Bacteroidota phylum) and the family *Saccharimonadaceae* (phylum Patescibacteria).

### Compositional Phylogenetic Shifts Characterized the Response of the Bacterial Communities to Flooding Stress

**FIGURE 6:**
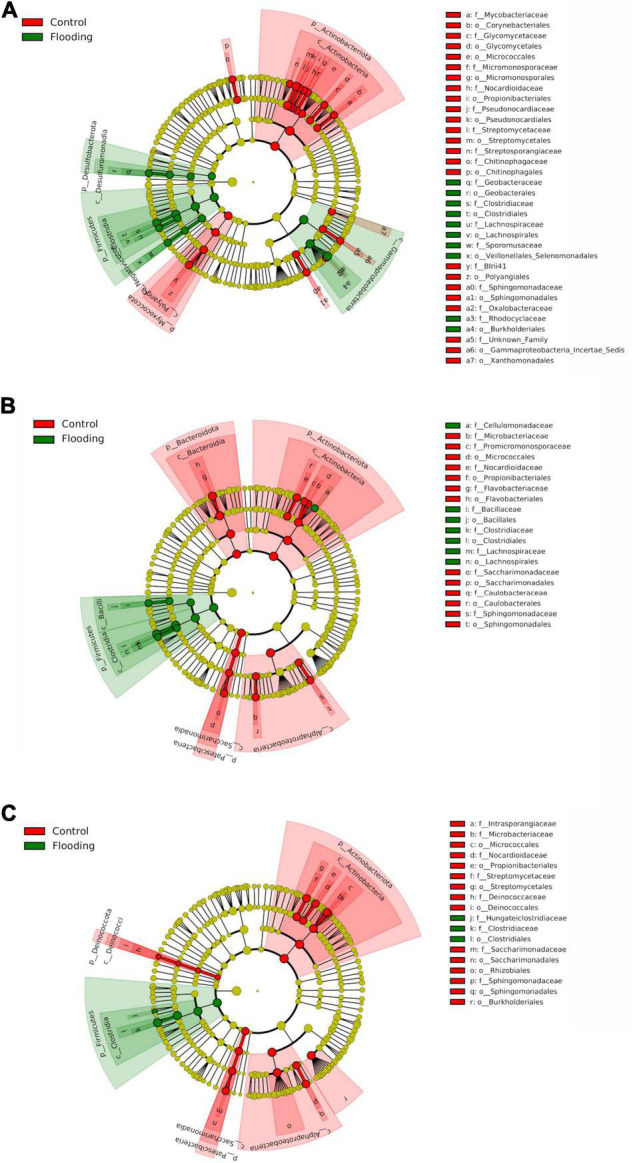
LEfSe analysis at multiple taxonomic levels comparing root bacterial community structure in both control and flooding treatment at tillering **(A)**, booting **(B)** and flowering **(C)**. Cladogram illustrating the taxonomic groups explaining the most variation among root communities. Each ring represents a taxonomic level, with phylum (p_), class (c_), order (o_) and family (f_) emanating from the center to the periphery. Each circle is a taxonomic unit found in the dataset, with circles or nodes shown in colors (other than yellow) indicating where a taxon was significantly more abundant.

We next investigated the composition and changes in relative abundance patterns of bacterial groups in response to this hydrological stress. The evident differences observed in bacterial beta-diversity between the flooded and control treatments were mainly reflected by substantial shifts in composition of the bacterial microbiota. For example, almost the half of the sequences that were detected in the flooded roots at tillering were not detected in the control treatment. These ASVs were mainly affiliated with the anaerobic or facultative anaerobic bacteria of the genera *Dechloromonas*, *Enterobacter*, *Geobacter* (Supplementary Figures 12, 13). Large proportions of unique ASVs were also only detected in flooding treatments in the roots at booting (38%) and flowering (20%) stages, which belonged mainly to the families *Clostridia, Oxalobacteraceae*, and *Lachnospiraceae.* These findings clearly demonstrated that flooding stress caused a greater disruption to early (tillering) compared to late (booting, flowering) bacterial microbiota, with a significant enrichment (*p* < 0.05) in facultative and strict anaerobes primarily of the class Clostridia (Firmicutes), and the phyla Desulfobacterota and Proteobacteria. LEfSe analysis further corroborated these observations, identifying in the root compartment almost twice of bacterial biomarker taxa at tillering than in the other PGS (Figure 6). More importantly, biomarker analysis evidenced that these compositional shifts of the bacterial microbiota due to flooding stress were highly phylogenetically clustered. Indeed, an enrichment of Firmicutes and Desulfobacterota together with a parallel depletion of Actinobacteriota and Proteobacteria, were observed in all the flooded samples. At the genus level, a significant increase (*p* < 0.05) in *Geobacter* and *Clostridium* abundances, with a parallel decrease of *Streptomyces* and *Sphinghomonas* were found.

Compartment-wise depletion and enrichment patterns at particular PGS were also recorded as a response to flooding. For instance, a significant decrease (*p* < 0.05) in the bacterial phyla Myxococcota at tillering, Bacteroidota at booting, Deinococcota at flowering, and Patescibacteria (especially the genus *Saccharimonadia*) in both late PGS was found in the flooded roots (Figure 6). On the contrary, a significant increase (*p* < 0.05) of Gammaproteobacteria occurred in all these samples. In particular, ASVs affiliated to Burkholderiales, such as (i) the endophytic non-nodulating diazotroph *Azoarcus*, and (ii) taxa with denitrifying and phosphorous accumulation capabilities of the genera *Dechloromonas* and *Candidatus Accumulibacter* decreased at tillering stage under flooding. In the rhizosphere, a general depletion of Rhizobiales, i.e., *Rhizobiaceae* and *Xanthobacteracee*, occurred due to flooding (Supplementary Figure 14). In addition, members of Cloroflexi, Verrucomicrobiota and Acidobacteriota where significantly depleted (*p* < 0.05) at tillering and booting, while an enrichment of Bacteroidota ASVs was detected in both tillering and flowering under flooding.

## Discussion

The plant microbiota is one of the key determinant for plant health and productivity (Turner et al., 2013; Finkel et al., 2017; Compant et al., 2019). Therefore, it is pivotal to unravel the factors influencing its composition and functionality in response to climate change-associated extreme weather events that likely will occur more frequently in the near future (Castro et al., 2010; De Vries and Shade, 2013). Herein, we provide an in-depth characterization of the effects flooding stress on the spring wheat microbiota complex.

### Flooding Causes a Joint and Substantial Change in Plant Phenotype and Root Microbiota

In our study, flooding reduced spring wheat performance as revealed by above- and belowground biomass changes, and significantly decreased the number of tillers and spike to tiller ratio, which can negatively affect the final plant productivity (Malik et al., 2002). Our findings are in accordance with previous studies since flooding have been reported to affect negatively plant performance, reducing root and shoot growth (Ghobadi et al., 2017), number of tillers and leaves (Arduini et al., 2016), photosynthesis (Jincai et al., 2001; Wu et al., 2015), kernel weight (Wu et al., 2015), and wheat yield (Pampana et al., 2016; Ding et al., 2020).

The wheat-microbiota was also strongly affected by hydrological stress, since the flooded and control bacterial communities where mainly composed by distinct taxa. These significant differences in community composition were correlated with changes in soil conditions and plant properties caused by flooding. Specifically, we identified pH and total N as the soil, and, S, Na, Mn, and Ca concentrations as the root properties most influencing microbial assemblage in the wheat microbiota under flooding stress (Supplementary Table 5). Beside O_2_ depletion, flooding strongly altered the soil physicochemical boundary conditions. It increased soil pH, C, and N, and influenced the concentration of several root macro- and micro-nutrients. Changes in soil pH is often reported as a consequence of waterlogging (Sun et al., 2007; Hemati Matin and Jalali, 2017) and it has been recognized as a main factor influencing the structure of bacterial microbiota across a wide range of soils and ecosystems (Fierer and Jackson, 2006; Lauber et al., 2009; Bardelli et al., 2017). Plant traits, such as root architecture and nutrient concentration are also commonly described as important drivers in structuring the root microbiota (Fitzpatrick et al., 2018; Schöps et al., 2018; Francioli et al., 2020), and their alterations might have important consequences in microbiota assembly (Leff et al., 2018; Pervaiz et al., 2020). Overall, these findings validated our first hypothesis, highlighting the fundamental influence of flooding on plant and soil properties, which in turn are firmly correlated with bacterial community assembly.

Our study further revealed that the changing root properties over the various PGS were important determinants for the assembly root-associated bacterial microbiota in both flooded and non-flooded wheat plants confirming previous observations that plant host phenological state plays a pivotal role in microbiota composition and structure (Bulgarelli et al., 2013; Wang et al., 2016; Francioli et al., 2018). In addition, a more severe impact of flooding on the bacterial microbiota occurred in the roots than in the rhizosphere suggesting that the root-associated community is under at a larger degree under host control. We found that the root microbiota showed a higher phylogenetically relatedness than the rhizospheric one, further highlighting the selective pressure exerted by plant host. It is noteworthy to mention that the two soil-plant compartments were characterized by distinct bacterial microbiota, with the root microbiota being a subset of the rhizospheric one. Collectively, these results proved that (i) niche compartmentalization plays a pivotal role in shaping microbiota in the soil-wheat system (Cordero et al., 2020; Tkacz et al., 2020; Kawasaki et al., 2021), and (ii) the root microbiota is highly controlled by host-factors that change over plant development (Lakshmanan, 2015; Lareen et al., 2016; Francioli et al., 2021b).

### The Wheat Microbiota Complex Is Less Resilient at Early Growth Stages to Flooding

Flooding stress caused dramatic shifts in microbiota composition and structure at each PGS in which it was induced, but it has the greatest impact on the bacterial community assembly at tillering stage. Phylogenetic analysis further showed that the bacterial microbiota in all the flooded samples associated with young plants were characterized by the lowest phylogenetic clustering similarities among the other bacterial community investigated in this study. These findings acknowledged our second hypothesis, as they proved that flooding caused a greater disruption to early compared with late PGS bacterial microbiota. Likewise, Xu et al. (2018) found that the juvenile sorghum root microbiota was more affected by drought stress compared to the one associated with late stages of plant development. In summary, all these observations suggest that the microbiota of early growth stages is still in a dynamic process of establishment, in which community assembly is less resilient to external physico-chemical stresses, while during the adult plant phase it is relatively more stable due to prior establishment of a more stable community likely with an higher and tighter degree of interactions (Angel et al., 2016; Edwards et al., 2018). The higher degree of compositional stability of bacterial communities in older wheat plants was also mirrored by the high phylogenetic community relatedness found in both flooding and control treatments at booting and flowering. Considering that flooding significantly affected the bacterial microbiota structure at all PGS, but no substantial differences were found in the phylogenetic alpha diversity metrics among flooded and control samples in older plants, we can deduce a general phylogenetic response of the bacterial microbiota to flooding stress in our wheat microbiota complex investigated, which was further confirmed by phylogenetic beta-diversity analysis using weighted UniFrac metric.

### Flooding Caused Shifts in the Phylogenetic Composition of the Bacterial Microbiota

The pattern of compositional shifts that flooding stirs a strong phylogenetic signal, with entire phyla responding roughly in unison, confirmed our third hypothesis. The main features of this pattern were (i) a significant increase of bacterial taxa with anaerobic respiratory capabilities, i.e., within phyla Firmicutes and Desulfobacterota, (ii) a significant reduction in Actinobacteria and the Proteobacteria, (iii) depletion of several putative plant-beneficial bacterial taxa by flooding stress, and (iv) increases of the abundance of potential detrimental taxa. Flooding promoted an enrichment of the genera *Geobacter* and *Clostridium*, that represent strictly anaerobic bacteria that are frequently isolated in waterlogged soils (Gschwend et al., 2020). Recent research showed that several *Clostridium* species might cause soft rot disease in several vegetable crops and their abundance significantly increased with heavy rainfall and flooding (da Silva et al., 2019). On the other hand, a dramatic reduction of the abundance of *Streptomyces* and *Spinghomonas* occurred in all flooding samples, which have been described as beneficial for wheat growth. Members of these two genera, are able to solubilize inorganic phosphorus, to form siderophores and affect phytohormones production and might be involved in biocontrol activity (Correa-Galeote et al., 2018; Kavamura et al., 2021). Specific compartment-wise patterns in enrichment or depletion of plant-health relevant bacteria in response to flooding were also detected. Bacteria affiliated to the genus *Saccharimonadia*, that recently was reported with putative beneficial features such as improving nitrogen uptake efficiency and promoting nutrient conversion, were mainly depleted in flooded roots (Herrmann et al., 2019; Dong et al., 2021). A similar trend was observed in the rhizosphere for families *Rhizobiaceae* and *Xanthobacteraceae* comprising multiple subgroups that might enhance and hinder plant development (Sadowsky and Graham, 1998; Oren, 2014). We also found that plant phenology was a significant and relevant factor shaping the bacterial community structure and differential responses to flooding were observed when such stress was induced at different PGS. Members of different *Massilia* species are considered as putatively plant-beneficial and frequently associated with wheat (Chimwamurombe et al., 2016). They produce proteases, sidephores and the auxin indole-3-acetic acid. *Massilia* ASVs were significantly more abundant in the control roots of young plants, but they were not detected in those of the flooded ones. Likewise, the flooded wheat roots at booting stage revealed a significant reduction of *Flavobacterium* ASVs, which are known for plant growth promoting traits. Bacteria of this genus have the capabilities to solubilize phosphate, use of 1-amino cyclopropan-1-carboxylate as sole nitrogen source and production of auxin, siderophores, salicylic acid, antifungal chitinases and hydrogen cyanide (Soltani et al., 2010; Verma et al., 2015; Gontia-Mishra et al., 2017). In summary, these findings demonstrated that flooding significantly altered negatively the assemblage dynamics of the root microbiota, with a significant depletion of putatively beneficial bacterial taxa associated with the root and rhizosphere of spring wheat plant.

It is important to note that the work undertaken here was limited to one soil type studied under very controlled greenhouse conditions. Further work should therefore investigate a greater range of soil types and flood scenarios, especially under realistic field conditions. Moreover, additional work is needed to fill the knowledge gaps in (i) how root exudation changes when crop species are faced with flooded growth conditions, (ii) how these exudates shape microbial community diversity and composition belowground, and (iii) the consequences for plant growth and functioning.

## Conclusion

This study illustrated the detrimental effect of flooding stress on the spring wheat-microbiota complex. Our findings demonstrated that such hydrological stress significantly reduced plant growth and fitness, together with dramatic changes in bacterial community assembly. Indeed, flooding significantly restructured spring wheat-microbiota composition and functionality, especially, in early plant development. In particular, flooding promoted an increase in the abundance of potential detrimental taxa, with a parallel reduction of putative plant-beneficial bacterial groups. These compositional shifts were primarily associated with profound alterations of edaphic and root properties, such as oxygen depletion, soil pH variation and changes in the concentration of several macro- and micro-nutrients in the rhizosphere and root compartment. Furthermore, our results revealed the pivotal role of plant phenology on the assemblage dynamics of the wheat root microbiota, since a differential response to flooding was also observed across the three PGSs. This emphasized the importance of temporal sampling when studying plant-associated microbiota, as they provide a more complete and robust picture of community response to environmental threats compared to the investigation of single time points. It is also noteworthy to mention that our study was only a beginning to explore the effect of flooding on wheat-microbiota complex, providing the baseline for future field experiment. Experiments under controlled laboratory conditions represent an essential starting point, but there is an urgent need to confirm insights from controlled study under realistic field conditions.

## Data Availability Statement

The datasets presented in this study can be found in online repositories. The names of the repository/repositories and accession number(s) can be found below: European Nucleotide Archive (ENA) under accession PRJEB47399 (ERP131670) https://www.ebi.ac.uk/ena/browser/view/PRJEB47399.

## Author Contributions

SKo, M-RH, and SKa planned the study. GC collected the samples. DF and GC performed the lab work, analyzed the data, and provided general guidance. DF wrote the manuscript. SKo, AU, M-RH, and GC contributed to reviewing the manuscript. All authors contributed to the article and approved the submitted version.

## Conflict of Interest

The authors declare that the research was conducted in the absence of any commercial or financial relationships that could be construed as a potential conflict of interest.

## Publisher’s Note

All claims expressed in this article are solely those of the authors and do not necessarily represent those of their affiliated organizations, or those of the publisher, the editors and the reviewers. Any product that may be evaluated in this article, or claim that may be made by its manufacturer, is not guaranteed or endorsed by the publisher.
