# Supplementary material for: Flooding Causes Dramatic Compositional Shifts and Depletion of Putative Beneficial Bacteria on the Spring Wheat Microbiota
Source: Front Microbiol. 2021 Nov 5;12:773116. doi: 10.3389/fmicb.2021.773116 (PMC8602104; doi:10.3389/fmicb.2021.773116)

Supplementary Material

**Flooding Causes Dramatic Compositional Shifts and Depletion of Putative Beneficial Bacteria on the Spring Wheat Microbiota**

Davide Francioli^1^*, Geeisy Cid^2^, Saranya Kanukollu^1^, Andreas Ulrich^1^, Mohammad-Reza Hajirezaei^2^ and Steffen Kolb^1,3^

^1^Microbial Biogeochemistry, Research Area Landscape Functioning, Leibniz Center for Agricultural Landscape Research e.V. (ZALF), Müncheberg, Germany

^2^Department of Physiology and Cell Biology, Leibniz Institute of Plant Genetics and Crop Plant Research, 06466 Gatersleben, Germany

^3^Thaer Institute, Faculty of Life Sciences, Humboldt University of Berlin, 10115 Berlin, Germany

*** Corresponding author***Davide Francioli
davide.francioli@zalf.de*

*Tel: +49 (0) 33432 82488*

**Table S1.** Bulk soil chemical properties of the soil used in the study.

| Parameter | Value | Mean standard error |
| --- | --- | --- |
| pH (KCl) | 5.42 | 0.003 |
| % TOC | 0.66 | 0.002 |
| % Nt | 0.07 | 0.001 |
| % St | 0.01 | 0.001 |
| NH4-N (mg/kg) | 0.78 | 0.003 |
| NO3-N (mg/kg) | 26.64 | 0.022 |
| PDL (mg/kg) | 60.80 | 0.011 |
| soil moisture | 3.43 | 0.232 |

**Table S2.** The relative importance of soil-plant compartment, plant growth stage (PGS) and watering treatment (WT) for the bacterial richness in the samples investigated in this study. Only interactions with significant effects are reported.

| Parameter | df | Pseudo-F | R^2^ | P‐value |
| --- | --- | --- | --- | --- |
| Compartment | 1 | 542.45 | 0.7270 | 0.001 |
| PGS | 2 | 1.27 | 0.004 | 0.286 |
| WT | 1 | 11.15 | 0.041 | 0.001 |
| Compartment * WT | 2 | 3.93 | 0.006 | 0.038 |
| PGS * WT | 2 | 5.02 | 0.106 | 0.001 |

**Table S3**. R values of pairwise comparisons of Bray-Curtis similarity between the different bacterial communities detected in the rhizospheric samples by ANOSIM analysis. Bold values indicate significant dissimilarity (P < 0.05) and are based on 999 permutations and Bonferroni corrections in all cases.

|  | Flooding-tillering | Control-tillering | Flooding-booting | Control-booting | Flooding-Flowering | Control-Flowering |
| --- | --- | --- | --- | --- | --- | --- |
| Flooding-tillering |  |  |  |  |  |  |
| Control-tillering | **1** |  |  |  |  |  |
| Flooding-booting | **0.994** | **0.859** |  |  |  |  |
| Control-booting | **1** | **0.818** | **0.814** |  |  |  |
| Flooding-Flowering | **0.988** | **0.937** | **0.818** | **0.853** |  |  |
| Control-Flowering | **0.992** | **0.801** | **0.859** | **0.570** | **0.640** |  |

**Table S4**. R values of pairwise comparisons of Bray-Curtis similarity between the different bacterial communities detected in the root samples by ANOSIM analysis. Bold values indicate significant dissimilarity (P < 0.05) and are based on 999 permutations and Bonferroni corrections in all cases.

|  | Flooding-tillering | Control-tillering | Flooding-booting | Control-booting | Flooding-Flowering | Control-Flowering |
| --- | --- | --- | --- | --- | --- | --- |
| Flooding-tillering |  |  |  |  |  |  |
| Control-tillering | **1** |  |  |  |  |  |
| Flooding-booting | **0.998** | **0.988** |  |  |  |  |
| Control-booting | **1** | **1** | **0.957** |  |  |  |
| Flooding-Flowering | **1** | **1** | **0.914** | **0.896** |  |  |
| Control-Flowering | **1** | **1** | **0.979** | 0.113 | **0.927** |  |

**Table S5.** Relationships between the predictor soil and plant variables and the bacterial community structure in the rhizosphere and roots.

|  | Rhizosphere | | Root | |
| --- | --- | --- | --- | --- |
|  | F | P | F | P |
| Soil pH | **2.562** | **0.001** | **2.362** | **0.006** |
| Soil N | **2.568** | **0.001** | **2.275** | **0.010** |
| Root S | **2.689** | **0.001** | **2.048** | **0.014** |
| Root K | ns | ns | **3.637** | **0.001** |
| Root Na | ns | ns | **5.051** | **0.001** |
| Root Mn | ns | ns | **1.835** | **0.029** |
| Root Ca | ns | ns | **1.696** | **0.046** |

Results show marginal tests using the db-RDA model. Significant P-values less than 0.05 are indicated in bold. ns, no significant

**FIGURE S1. Experimental design of the greenhouse experiment conducted in this study.**

**
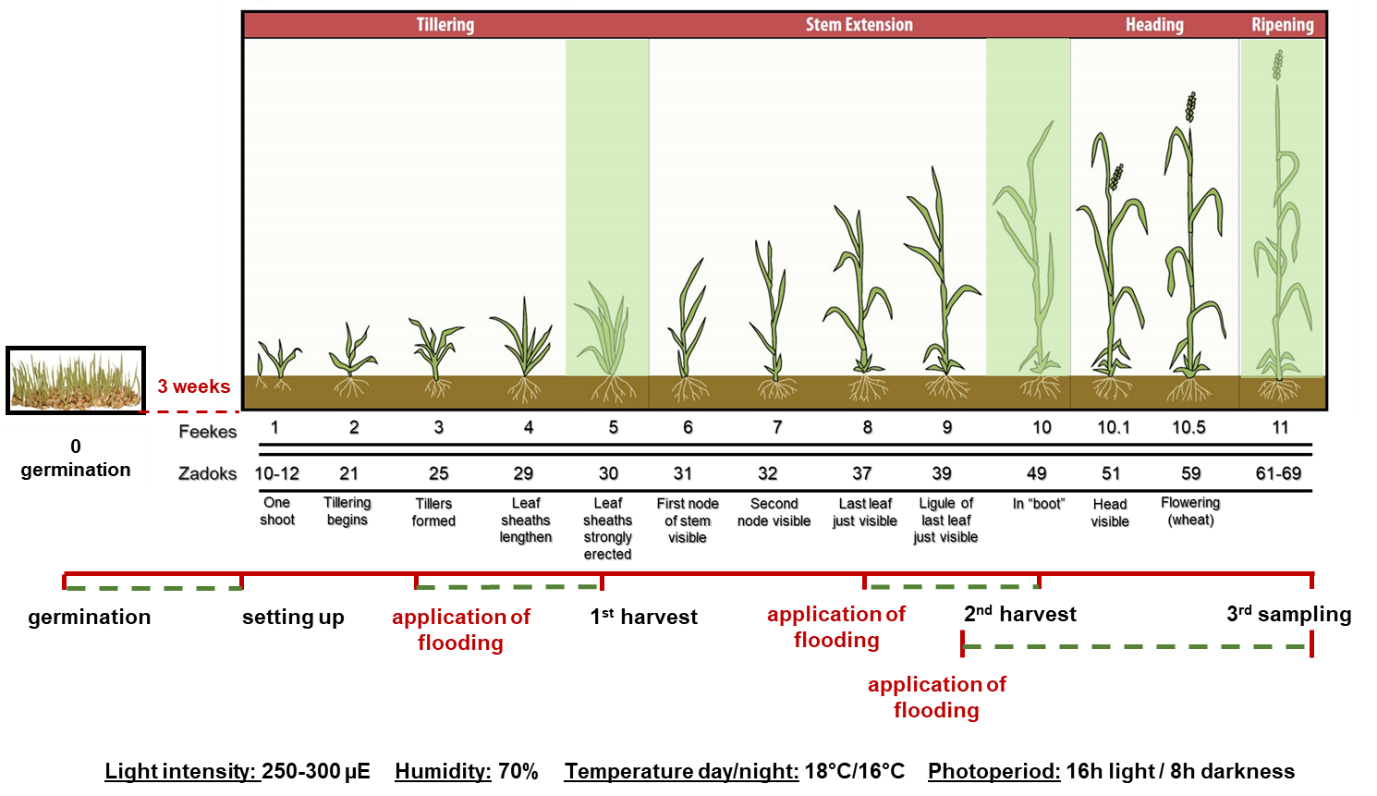
**

**FIGURE S2. Shoot (A) and root (B) dry biomass, numbers of tiller (C) and spike tiller ratio (D) detected in the control and flooded samples.** The different letters indicate significant differences within plant growth stages (Tukey’s HSD test *p* < 0.05.).

**
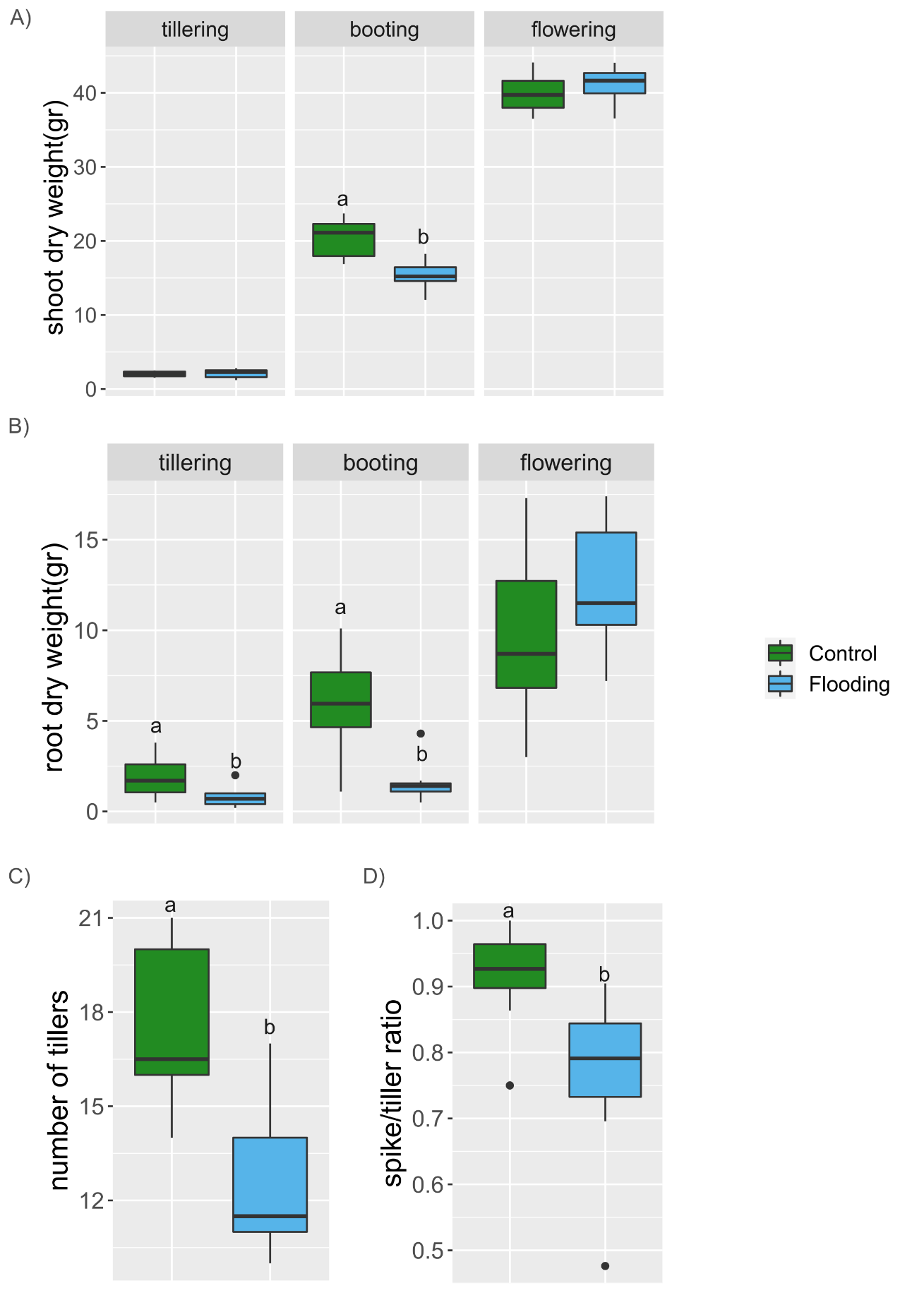
**

**FIGURE S3. Soil parameters measured in the different water treatments and PGS.** The different letters indicate significant differences within plant growth stages (Tukey’s HSD test *p* < 0.05.).

**
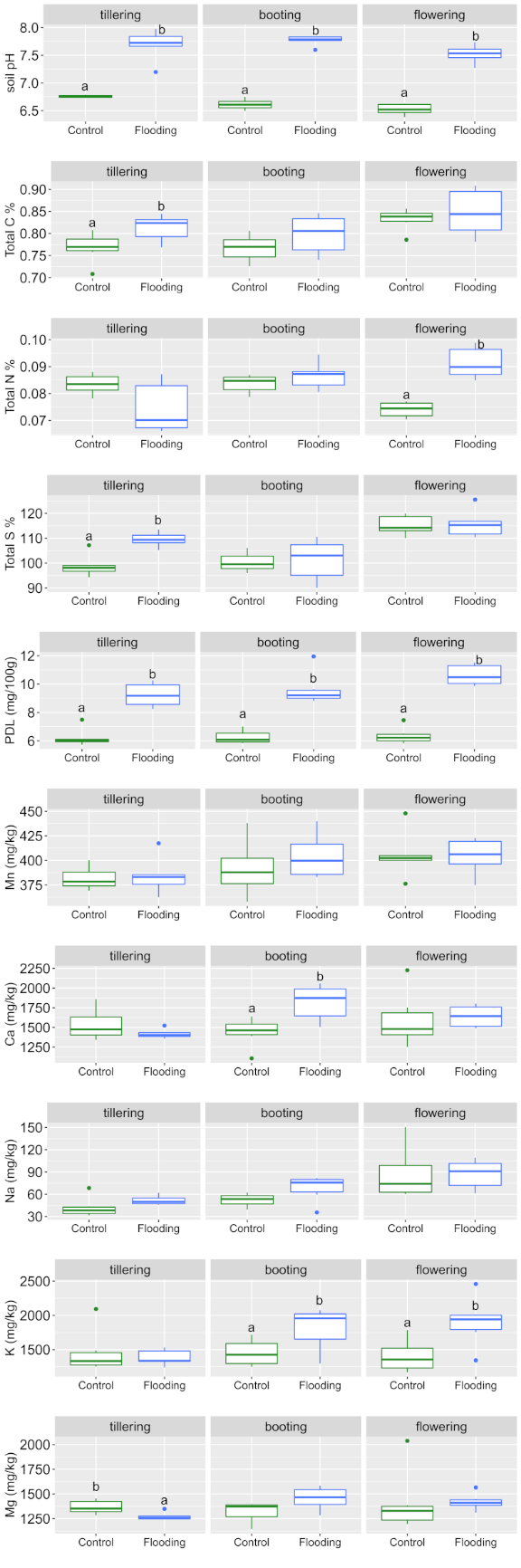
**

**FIGURE S4. Root traits measured in the different water treatments and PGS.** The different letters indicate significant differences within plant growth stages (Tukey’s HSD test *p* < 0.05.).

**
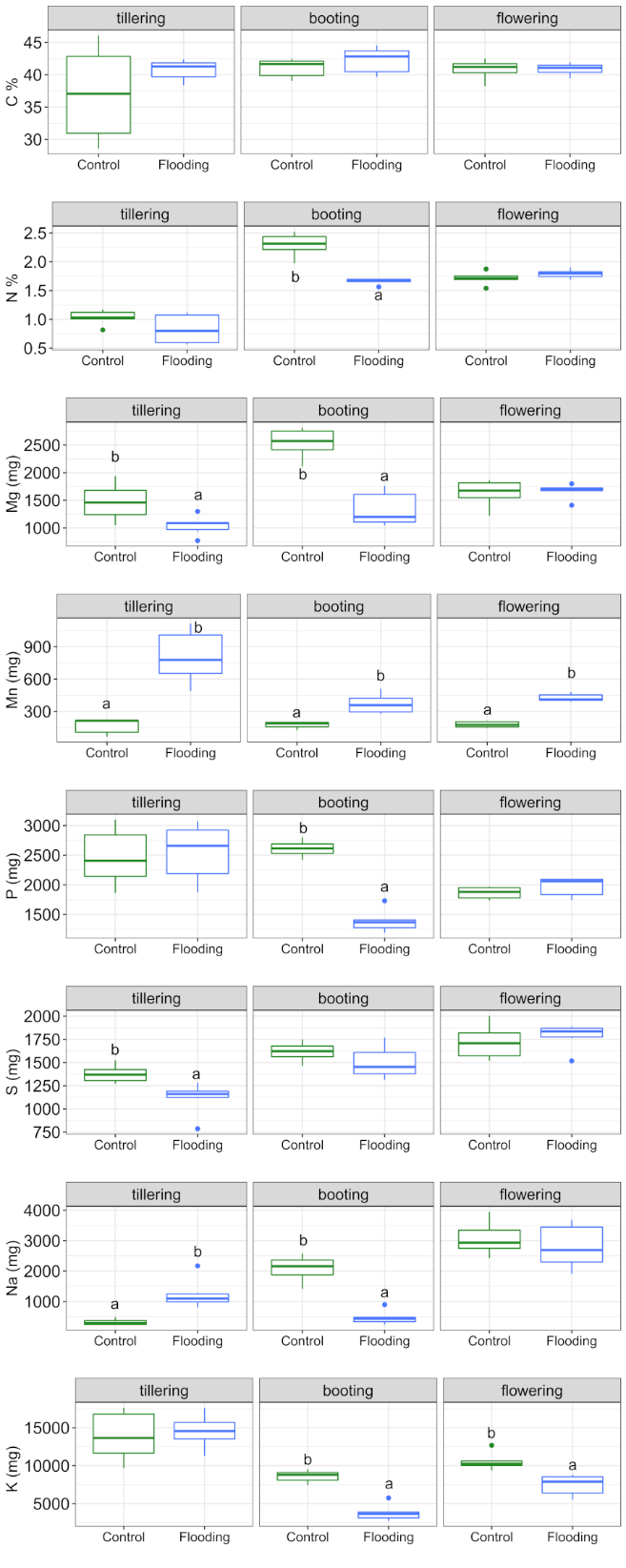
**

**FIGURE S5. Boxplots describing the net relatedness index (NRI) (A) and the nearest taxa index (NTI) (B) in the different compartments, plant growth stages and watering treatments investigated in this study.** Different letters indicate significant differences between water treatments within plant growth stages based on Tukey’s HSD test *p* < 0.05.

**
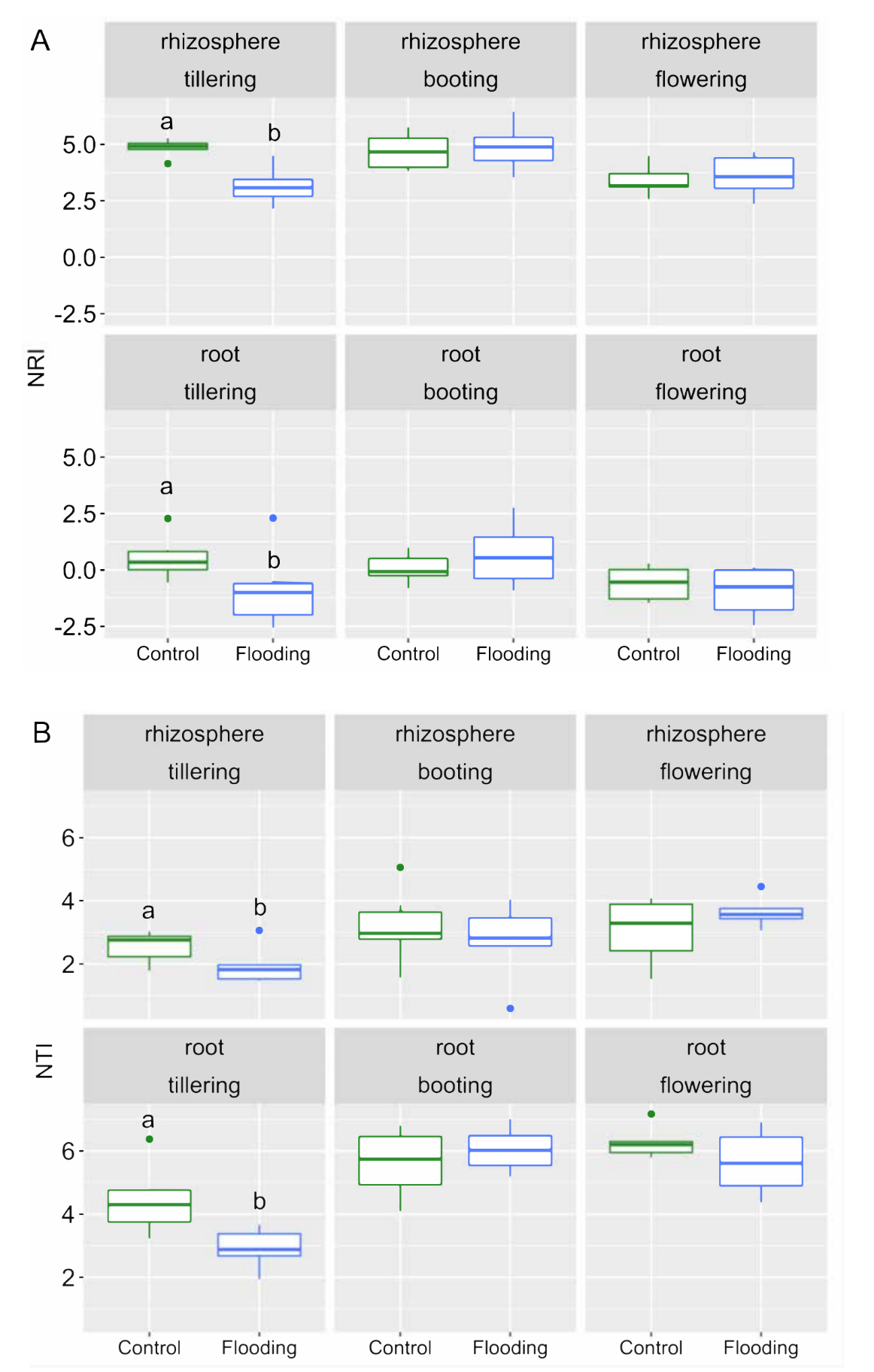
**

**FIGURE S6.** **Principal Coordinates Analysis (PCoA) using weighted UniFrac distance of the bacterial communities detected in this study.**


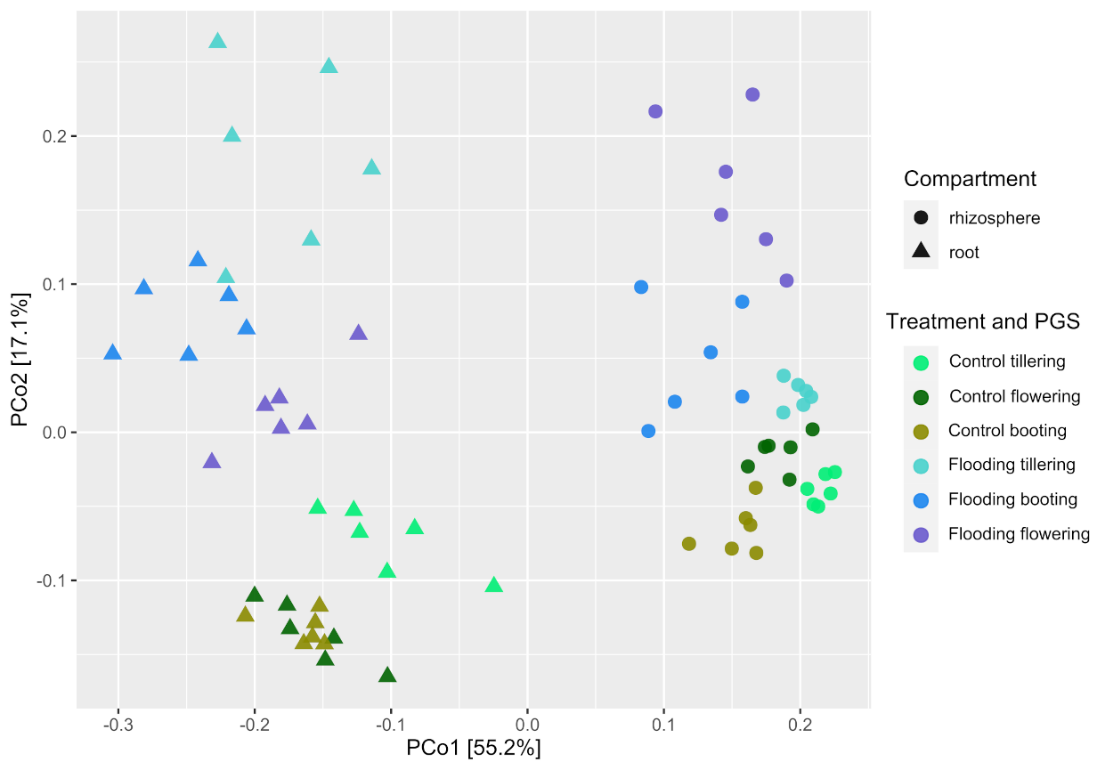


**FIGURE S7. Relative abundances of the main phyla in the rhizospheric and root samples (A). Venn diagram of the shared and unique ASVs detected in the rhizosphere and roots (B).**

**
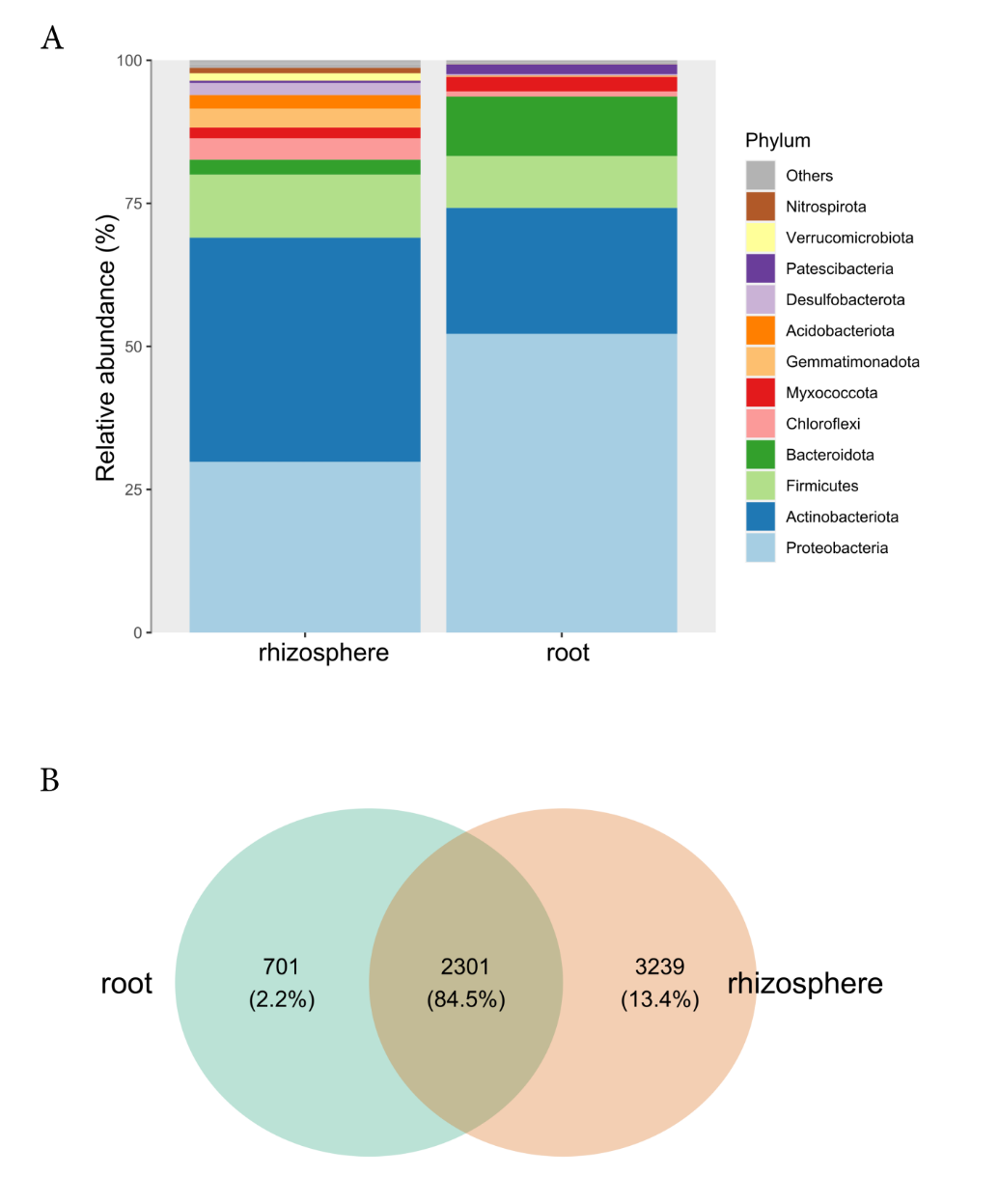
**

**FIGURE S8.** **Principal Coordinates Analysis (PCoA) using Bray-Curtis dissimilarity metrics of the bacterial communities of the (A) rhizosphere and (B) roots associated with the control treatment.**

**
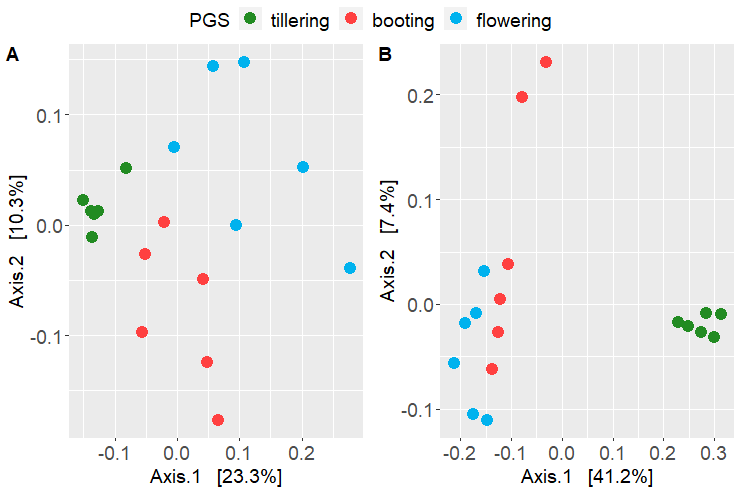
**

**FIGURE S9. Variation partitioning analysis, illustrating the effects soil parameters, root traits and plant growth stage (PGS) in the community for the rhizosphere (A) and root (B) compartment of the control plants.** Each ellipse represents the portion of variation accounted by each factor. Shared variance is represented by the intersecting portions of the ellipses. Values <0 are not shown. Unexplained variation (residuals) is reported in the plot.


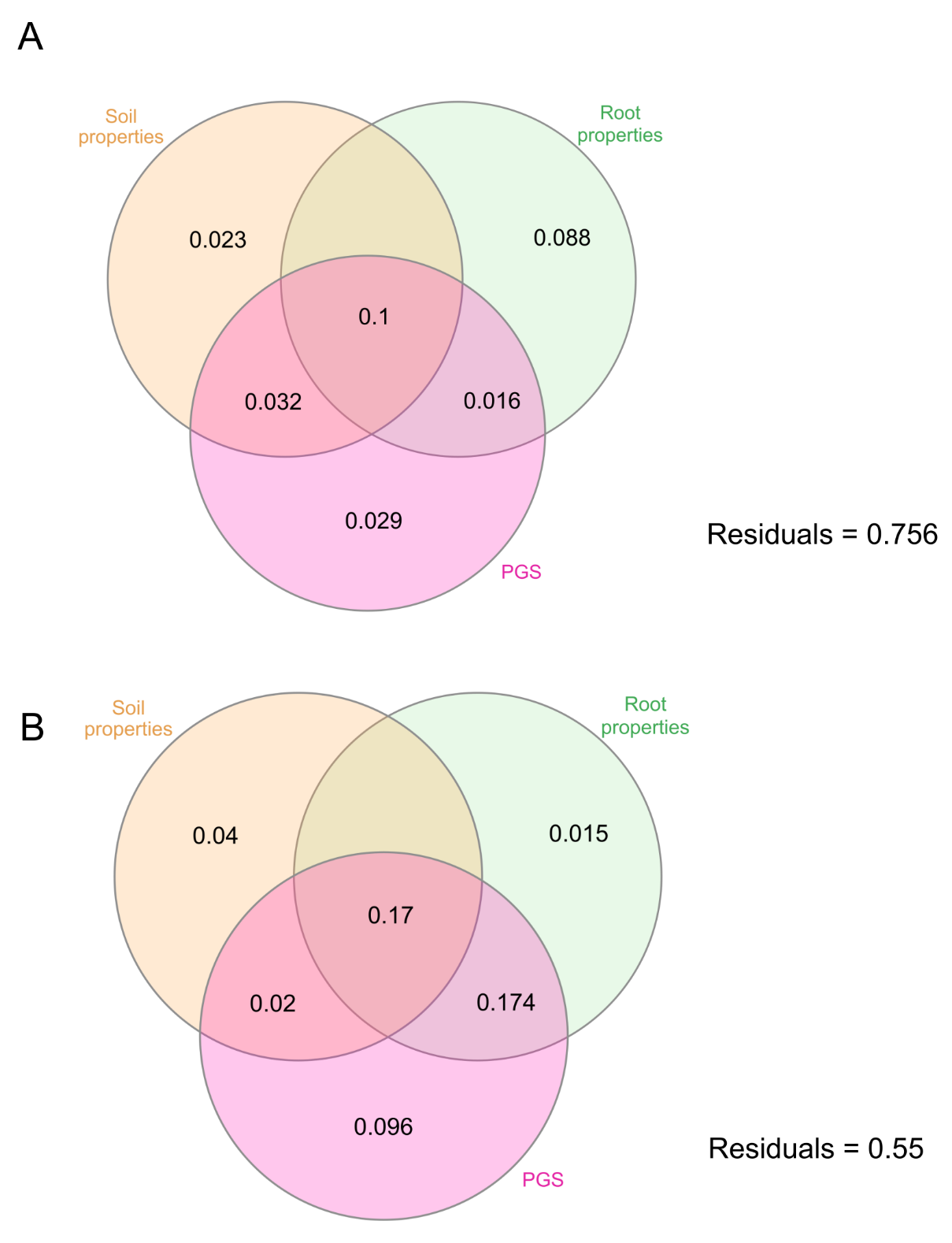


**FIGURE S10. Venn diagrams showing the unique and shared ASVs at each PGS for the the rhizospheric (A) and root (B) communities. Relative abundances of the ASVs that are shared across each PGS at the genus (g__) or family (f__) level in the rhizosphere (C) and in the root (D) compartment. Only the taxonomic clades (genera and families) that account for abundance >1% are displayed.**

**
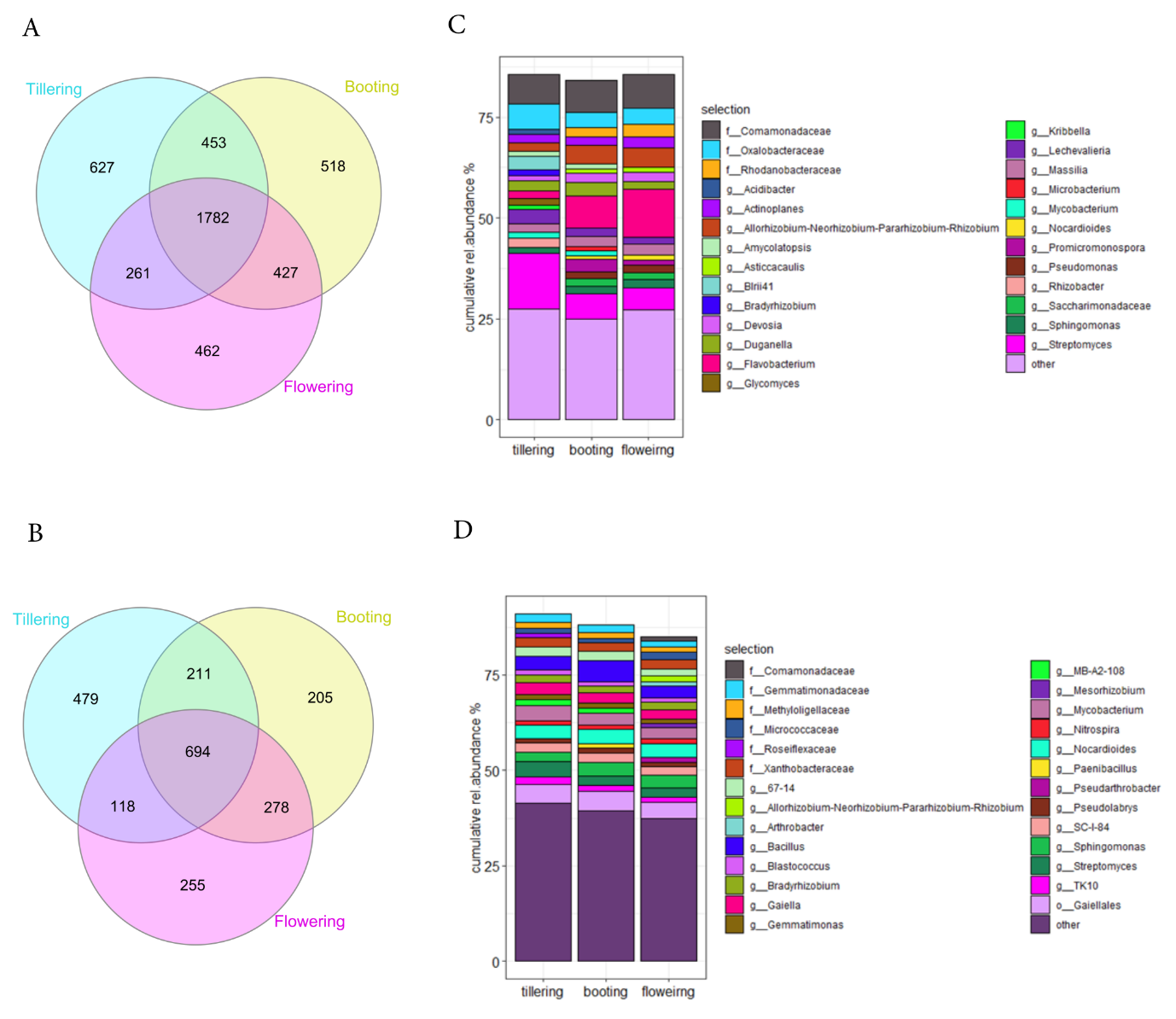
**

**FIGURE S11. LEfSe analysis at multiple taxonomic levels comparing rhizospehric (A) and root (B) bacterial community structure in control samples across the three PGS. Cladogram illustrating the taxonomic groups explaining the most variation among the bacterial communities. Each ring represents a taxonomic level, with phylum (p), class (c), order (o), family (f) and genus (g) emanating from the center to the periphery. Each circle is a taxonomic unit found in the dataset, with circles or nodes shown in colors (other than yellow) indicating where a taxon was significantly more abundant.**

**
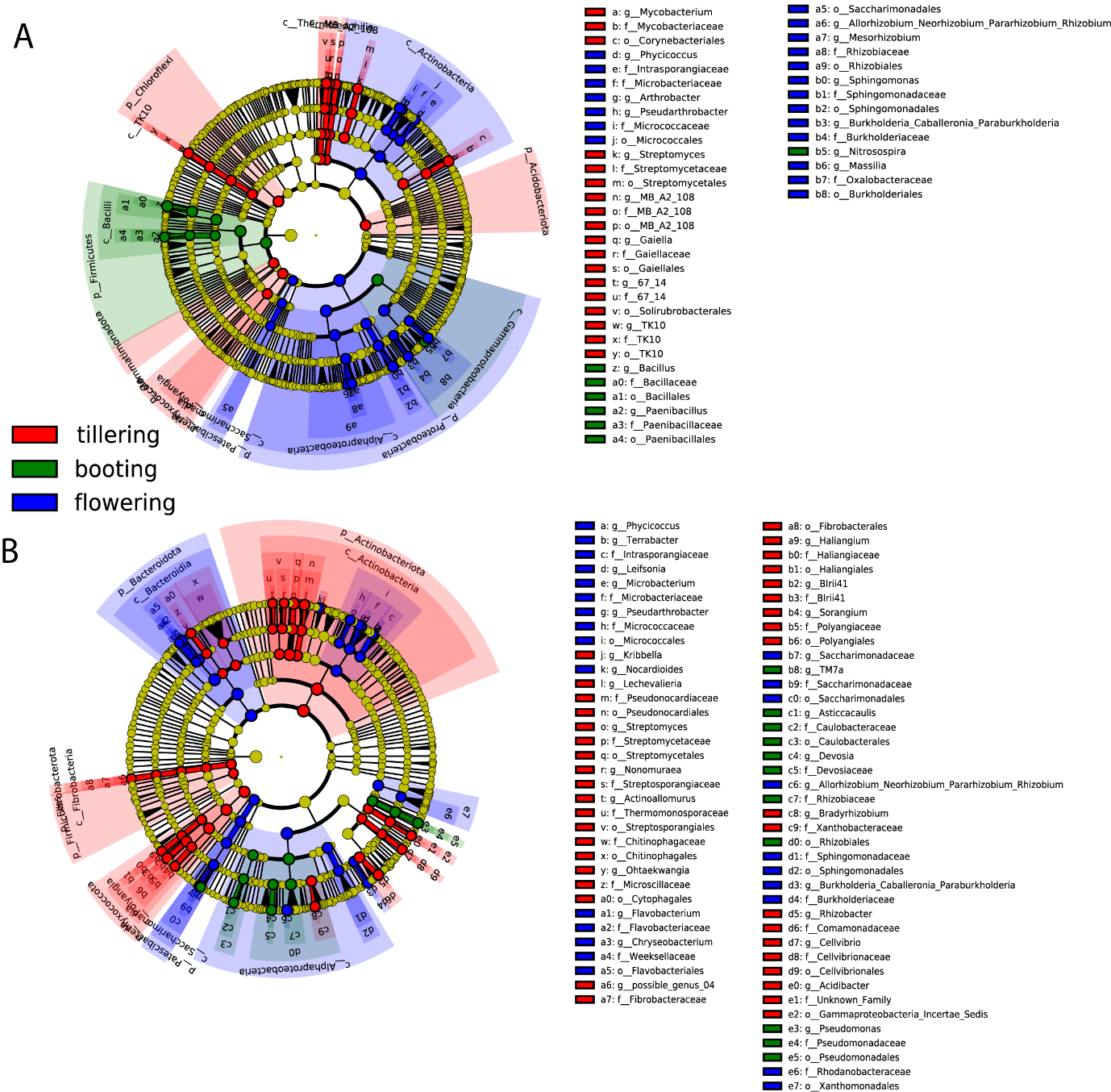
**

**FIGURE S12. Venn diagrams showing the unique and shared ASVs between control and flooded rhizospheric communities at tillering (A), booting (B) and flowering (C). Relative abundances of the ASVs that are unique for each watering treatment clustered at the genus (g__) or family (f__) level at tillering (D), booting (E) and flowering (F). Only the taxonomic clades (genera and families) that account for abundance >1% are displayed.**


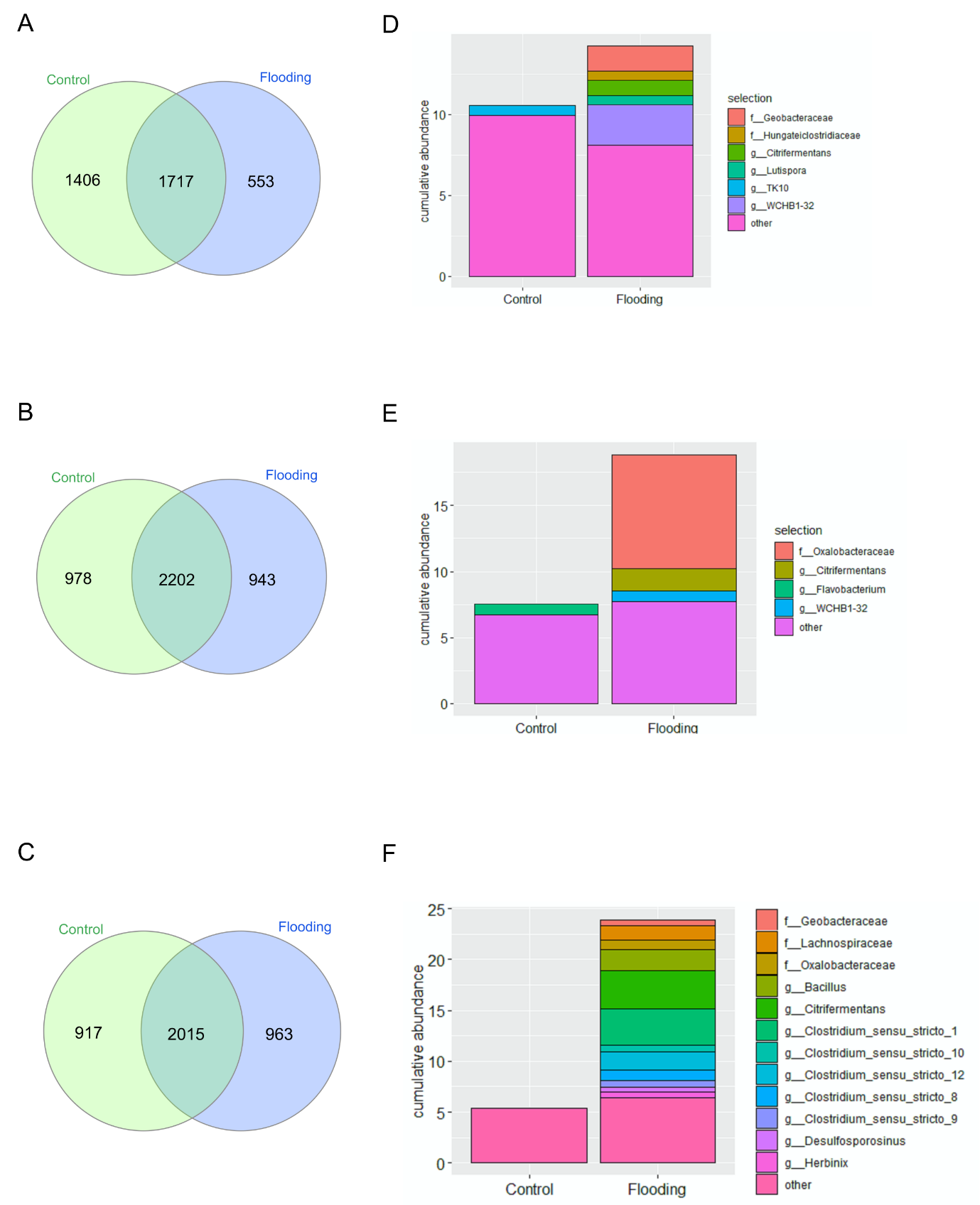


**FIGURE S13. Venn diagrams showing the unique and shared ASVs between control and flooded root communities at tillering (A), booting (B) and flowering (C). Relative abundances of the ASVs that are unique for each watering treatment clustered at the genus (g__) or family (f__) level at tillering (D), booting (E) and flowering (F). Only the taxonomic clades (genera and families) that account for abundance >1% are displayed.**

**
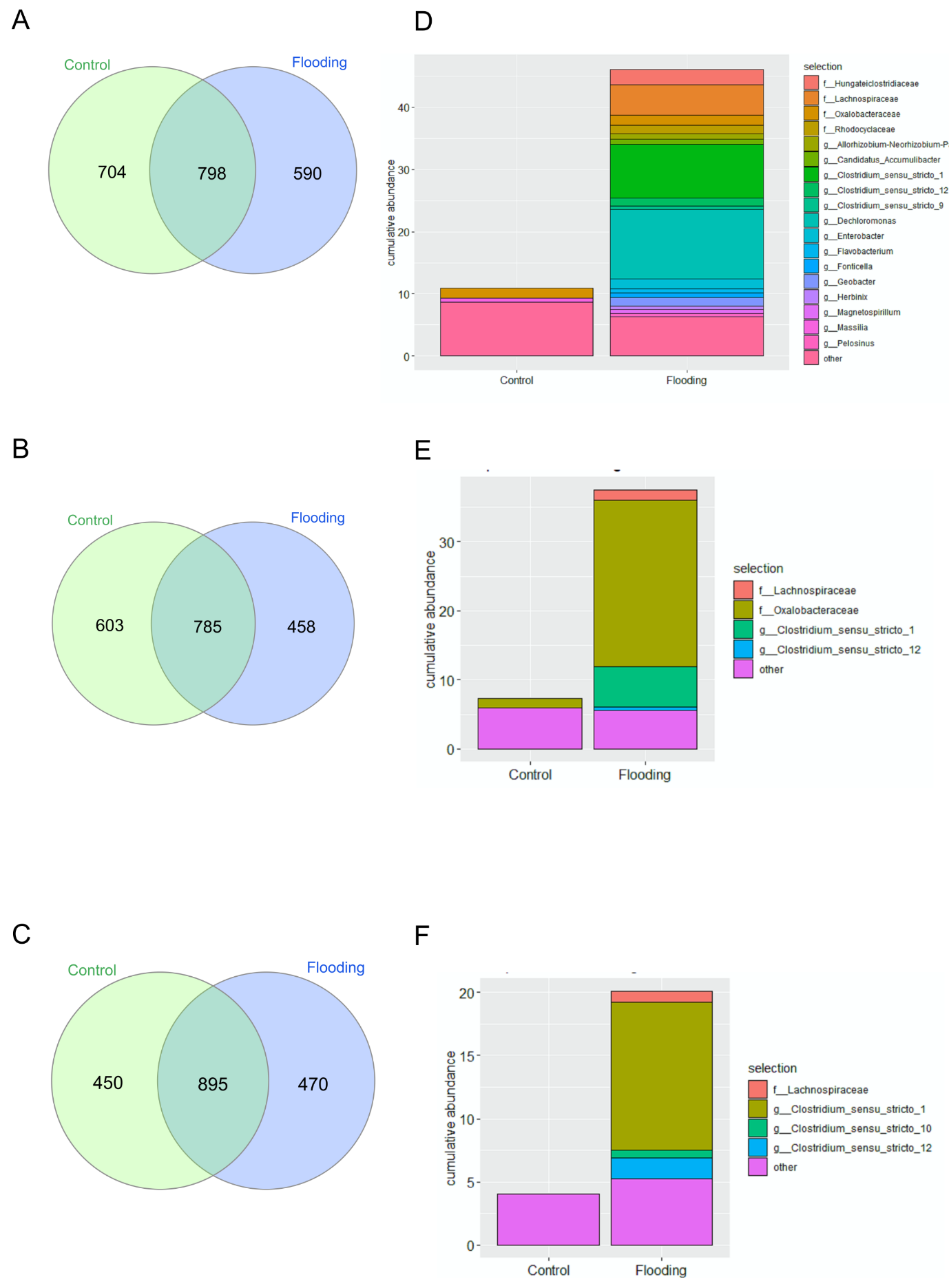
**

**FIGURE S14. LEfSe analysis at multiple taxonomic levels comparing rhizospheric bacterial community structure in both control and flooding treatment at tillering (A), booting (B) and flowering (C). Cladogram illustrating the taxonomic groups explaining the most variation among root communities. Each ring represents a taxonomic level, with phylum (p), class (c), order (o), family (f) and genus (g) emanating from the center to the periphery. Each circle is a taxonomic unit found in the dataset, with circles or nodes shown in colors (other than yellow) indicating where a taxon was significantly more abundant.**


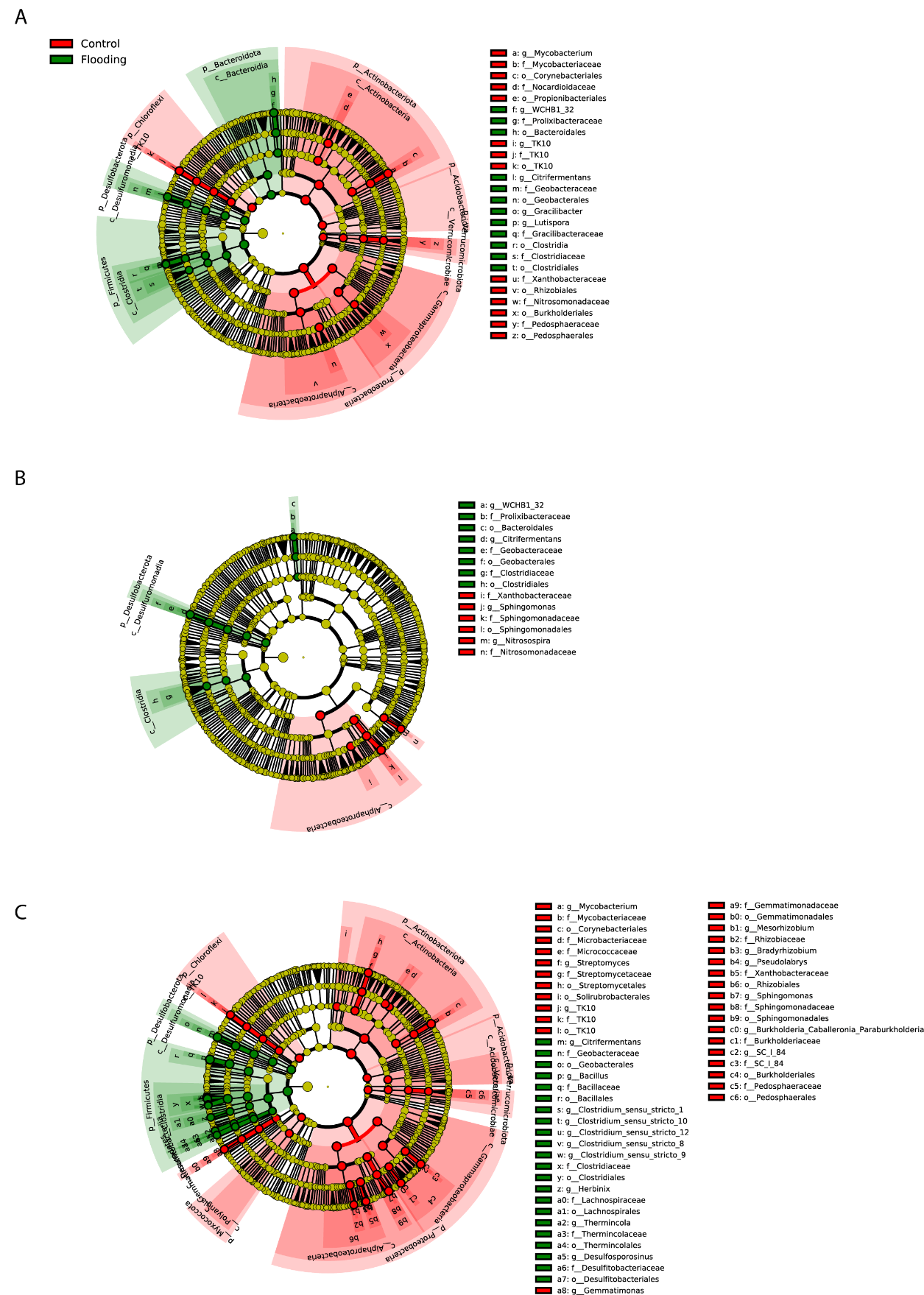

Supplement: Supplementary file 1 [file Data_Sheet_1.docx]
